# Identification and ultra‐high‐performance liquid chromatography coupled with high‐resolution mass spectrometry characterization of biosurfactants, including a new surfactin, isolated from oil‐contaminated environments

**DOI:** 10.1111/1751-7915.13276

**Published:** 2018-05-14

**Authors:** Glaci V. Moro, Rafaela T.R. Almeida, Amanda P. Napp, Carla Porto, Eduardo J. Pilau, Diogo S. Lüdtke, Angélica V. Moro, Marilene H. Vainstein

**Affiliations:** ^1^ Centro de Biotecnologia Universidade Federal do Rio Grande do Sul (UFRGS) Av. Bento Gonçalves 9500 91501‐970 Porto Alegre RS Brazil; ^2^ Departamento de Química Universidade Estadual de Maringá (UEM) 87020‐900 Maringá PR Brazil; ^3^ Institute of Chemistry Universidade Federal do Rio Grande do Sul (UFRGS) Av. Bento Gonçalves 9500 91501‐970 Porto Alegre RS Brazil

## Abstract

Biosurfactant‐producing bacteria were isolated from samples collected in areas contaminated with crude oil. The isolates were screened for biosurfactant production using qualitative drop‐collapse test, oil‐spreading and emulsification assays, and measurement of their tensoactive properties. Five isolates tested positive for in the screening experiments and displayed decrease in the surface tension below 30 mN m^−1^. The biosurfactants produced by these isolates were further investigated and their molecular identification revealed that they are bacteria related to the *Bacillus* genus. Additionally, the biosurfactants produced were chemically characterized via UHPLC‐HRMS experiments, indicating the production of surfactin homologues, including a new class of these molecules.

## Introduction

A variety of remediation strategies have surfaced as a way to minimize the damage created by the liberation of hydrocarbons onto the environment. In this context, a number of cleaning strategies have been developed for oil‐contaminated water; the two most frequent being physical removal and the use of chemical dispersants, also known as surfactants. Surfactants are amphiphilic molecules with both hydrophilic and hydrophobic moieties; they display emulsification abilities and also reduce the surface tension between two fluids of different polarities (Banat *et al*., 2010; Franzetti *et al*., 2010; Hassanshahian, 2014). However, these compounds are not biodegradable and can be toxic to the environment. An alternative solution to this problem is the use of biosurfactants, which are produced by a wide range of different microorganisms through relatively cheap fermentation processes using sugars or vegetal oils as carbon sources. Biosurfactants have received growing attention due to their advantages over synthetic surfactants, these include biodegradability, low water toxicity and higher tolerance levels to temperature, pH and salt concentration (Yang *et al*., 2015). Biosurfactants can be chemically classified into low‐molecular‐weight compounds (e.g. glycolipids and lipopeptides) and high‐molecular‐weight compounds (e.g. polysaccharides, proteins and lipoproteins) (Zhou *et al*., 2015). Amongst the most efficient biosurfactant‐producing microorganisms are those from the genus *Bacillus*, mostly due to its ability to produce cyclic lipopeptides bearing a long acyclic side‐chain. The three main families of lipopeptides produced by *Bacillus* spp. are surfactins, iturins and fengycins (Geissler *et al*., 2017). Each of these families of lipopeptides consists of several congeners, which differ either in the length of the fatty acid side‐chain or in the amino acid sequence at the cyclic peptide (Jacques, 2011).

Biosurfactants have been used in a number of environmental applications, particularly in the bioremediation of oil spills and in the removal of residual oil from storage tanks (Sousa *et al*., 2012). Additionally, biosurfactants have potential uses as emulsifiers in the food, cosmetic and pharmaceutical industries. The surfactin lipopeptide is known for its antiviral, antibacterial and antitumoral properties, while iturins and fengycins display antifungal properties (Malfanova *et al*., 2012; Meena and Kanwar, 2015).

Considering the importance of the discovery of new and efficient biosurfactant‐producing organisms, our results regarding the isolation, identification and characterization of biosurfactants by the screening of microorganisms isolated from oil‐contaminated environments are herein outlined (Gudiña *et al*., 2012). A detailed study to characterize the chemical composition of the biosurfactants produced was carried out. Ultra‐high‐performance liquid chromatography coupled with high‐resolution mass spectrometry (UHPLC‐HRMS) revealed the production of a series of surfactin homologues, including a new class of these biosurfactants.

## Results and Discussion

### Screening of microorganisms for the production of the biosurfactants

Two hundred microorganisms isolated from oil‐contaminated soil samples obtained from different Brazilian terrestrial sites were used in this study. These microorganisms were subjected to liquid fermentation using 5% glucose as the only carbon source, to evaluate their biosurfactant‐producing ability. Crude oil was also used as the carbon source in preliminary experiments. However, only poor production of biosurfactants was observed, even in longer incubation periods. Therefore, to assess whether the microorganism had the ability to produce biosurfactants or not, the faster and more efficient growing method, using glucose as the carbon source, was chosen.

The drop‐collapse test and oil‐spreading assay are qualitative and easy‐to‐read tests which were used for preliminary evaluation of the ability of a given microorganism to produce a biosurfactant. Initial screening revealed that 14 out of 200 microorganism isolates tested positive for both assays (Table 1). *Pseudomonas aeruginosa* ATCC 27853 was used as the positive control. As the negative control, a sample of the MSM supplemented with 5% glucose was used.

**Table 1:** Biosurfactant production by microorganisms isolates cultivated in MSM with 5% glucose as the only carbon source

Isolates	Origin	Drop‐collapse test^c^	Oil‐spreading test^c^
Negative control^a^	–	–	–
Positive control^b^	UFRGS	+++	+++
BPB 1.6	UFRGS	++	++
ODW02	ODW	+++	++
ODW109	ODW	++	++
ODW115	ODW	++	+
ODW12	ODW	+++	++
ODW123	ODW	++	+
ODW15	ODW	++	++
ODW16	ODW	++	+
ODW04	ODW	++	+
MO13	UFRGS	+++	+++
MO4A	UFRGS	++	++
MO4B	UFRGS	+++	+++
BPB 1.18	UFRGS	++	++
MO74	UFRGS	++	++

a Negative Control = MSM medium with 5% glucose.

b Positive control = *P. aeruginosa* ATCC 27853.

c Symbol means: (−) no results; (+) average results; (++) good results (+++) excellent results.

### Tensoactive properties of biosurfactants

The 14 isolates were then subjected to the emulsification assay (E_24_) and measurement of the surface tension (Table 2). It is worth pointing out that despite displaying good results in the drop‐collapse and oil‐spreading assays, some of the isolates did not perform well in the emulsification assay. For example, negative results were observed with ODW109, ODW115, ODW16, ODW4A and *P. aeruginosa*, while MO13 and ODW04 have shown low emulsification ability. It should be mentioned, however, that these tests are used to measure different properties of tensoactive compounds. The remaining eight isolates have displayed excellent results, with E_24_ in the range of 65.7–80.5%, values that are higher than the 40%, which is considered satisfactory (Youssef et al., 2004).

**Table 2:** Measurements of emulsification index (E_24_) and surface tension of culture after growth of isolates in minimal medium with 5% glucose as the carbon source for the production of biosurfactants. The results for water, ethanol, negative control and positive control (*Pseudomonas aeruginosa*) are also shown

Isolates	E_24_ (%)^a^	Surface tension (mN m^−1^)^a^
Negative control	0	70.7 ± 0.09
Positive control	0	28.4 ± 0.16
Water	Not applicable	71.8 ± 0.25
Ethanol	Not applicable	23.0 ± 0.11
BPB 1.6	71.8 ± 3.12	34.9 ± 0.58
ODW02	80.5 ± 8.33	28.8 ± 0.00
ODW109	0	34.2 ± 0.16
ODW115	0	33.9 ± 0.08
ODW12	77.7 ± 5.55	28.7 ± 0.41
ODW123	66.8 ± 6.84	38.7 ± 0.64
ODW15	68.3 ± 1.66	28.4 ± 0.00
ODW16	0	31.7 ± 0.70
ODW04	34.3 ± 9.37	34.8 ± 1.23
MO13	36.1 ± 2.77	27.1 ± 0.64
MO4A	0	35.2 ± 0.95
MO4B	65.7 ± 0.98	27.6 ± 1.05
BPB 1.18	72.2 ± 5.55	32.2 ± 0.86
MO74	71.6 ± 3.40	31.9 ± 0.10

a Values reported are averages of three replicates ± the standard error.

A decrease in the surface tension measurements was observed in the selected isolates, in comparison with pure water and the negative control. For example, five isolates (ODW02, ODW12, ODW15, MO13 and MO4B) and the positive control (*P. aeruginosa*) have displayed surface tensions below 30 mN m^−1^, which are exceptional results when compared to existing literature, which indicates that a surface tension value of 35 mN m^−1^ is required for an efficient biosurfactant (Patowary *et al*., 2015). All tests were performed with cell‐free supernatants. This is an important feature, as isolates that liberate biosurfactants into the culture medium render the recovery process simpler, making them more interesting from an industrial perspective (Kuyukina *et al*., 2001; Batista *et al*., 2006).

Despite the negative result displayed by the *P. aeruginosa* supernatant in the emulsification assay, other tensoactive properties experiments such as oil spreading, drop collapsing and surface tension experiments tested positive and the production of biosurfactants was confirmed. Similar behaviour was also observed by other authors (Belgacem *et al*., 2015). It is known from the literature (Christova *et al*., 2011; Ndlovu *et al*., 2017; Nicolò *et al*., 2017) that several growing factors such as the carbon source, specific culture conditions, age of culture and the *Pseudomonas* strain influence the composition of the rhamnolipid biosurfactant produced by *Pseudomonas,* influencing the emulsification activity.

The isolates that produced biosurfactants which displayed surface tensions below 30 mN m^−1^ (ODW15, ODW12, ODW02, MO4B and MO13) were selected for molecular identification, chemical characterization of the biosurfactants produced, determination of their critical micelle concentration (CMC) and chemical stability.

### Molecular identification

Molecular identification of the five selected isolates was performed based on the 16S rRNA gene sequences, using the GenBank BLAST tool. It was found that all of the microorganisms were closely related to the *Bacillus* genus. The isolates were related to the following species: ODW15: *Bacillus subtilis* (99%), ODW12: *Bacillus gibsonii* (98%), ODW02: *Bacillus subtilis* (99%), MO4B: *Bacillus amyloliquefaciens* (99%) and MO13: *Bacillus amyloliquefaciens* (99%). It is worth pointing out that only the best‐performing microorganisms have been molecularly characterized. From the entire panel studied, microorganisms that were not *Bacillus* have been identified. However, as those did not perform well as biosurfactant producer, further efforts for a more detailed characterization were not pursued.

### Chemical characterization of the biosurfactants

The chemical biosurfactant characterization of isolates *B. subtilis* ODW15, *B. gibsonii* ODW12, *B. subtilis* ODW02, *B. amyloliquefaciens* MO4B and *B. amyloliquefaciens* MO13 was performed using TLC and UHPLC‐HRMS techniques. For the characterization of the biosurfactants, the supernatants of the bacterial cultures were initially extracted with chloroform/methanol, yielding brown oils after the removal of the organic solvent. TLC analysis was performed using ethyl acetate as the eluent and the plates were visualized; ninhydrin was used as a developing agent for the detection of peptides, anisaldehyde staining for the detection of carbohydrates and anthrone for the detection of reducing sugars. The TLC analysis suggested that the biosurfactants produced were peptides, as they developed colour when ninhydrin was used (Smyth *et al*., 2010). When the developing agent anthrone was used, a blue colour was not observed in any of the samples. However, the positive control *P. aeruginosa*, known for the production of rhamnolipids, showed a blue colour when exposed to anthrone. Altogether, these findings further support the view that the biosurfactants produced are not glycolipids, but are instead lipopeptides.

### High‐resolution mass spectrometry studies

**Figure 1:**
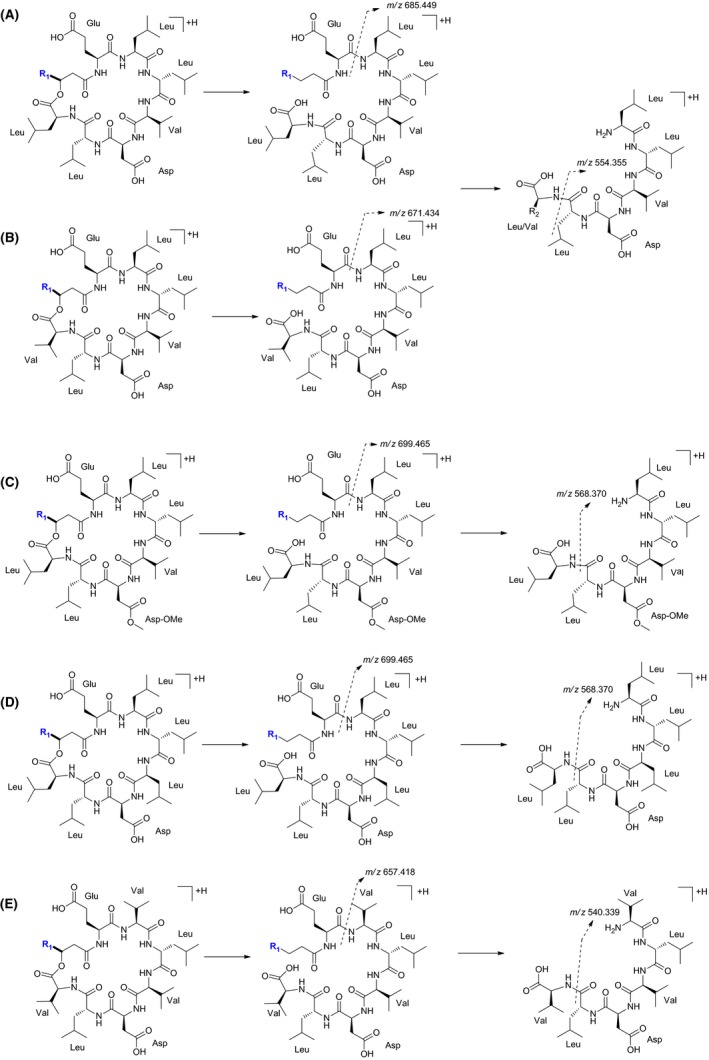
Fragmentation pattern for surfactin isoforms. (A) Surfactin A, fragment ions with m/z 685.449 and 554.355. (B) Surfactin B, fragments ions with *m/z* 671.434 and 554.355. (C) Surfactin monomethyl ester, fragments ions with *m/z* 699.465 and 568.370. (D) [Leu4]surfactin, fragments ions with *m/z* 699.465 and 568.370. (E) Isoform surfactin, fragments ions with m/z 657.418 and 540.339. Groups R1: (CH2)5‐11CH(CH3)2.

To fully elucidate the structures of the biosurfactants produced, UHPLC‐HRMS studies have been performed, and these studies revealed the presence of surfactins (Fig. 1). The surfactin family consists of a mixture of surfactin isoforms, in which an amino acid of the cyclic peptide chain of surfactin Glu/Leu/Leu/Val/Asp/Leu/Leu, is replaced by another amino acid (Peypoux *et al*., 1994). These isoforms were differentiated according to their fragmentation pattern and structural features (Fig. 1A–E). The MS/MS spectra of surfactins isoforms presented common fragments [M+H]^+^ at *m/z* 685.44 (Fig. 1A), 671.43 (Fig. 1B), 699.46 (Fig. 1C) and 699.46 (Fig. 1D). These ions are from characteristic amino acid sequences, previously reported as Val/Leu/Asp/Val/Leu/Leu, Leu/Leu/Asp/Val/Leu/Leu, Leu/Leu/Asp‐OMe/Val/Leu/Leu and Leu/Leu/Asp/Leu/Leu/Leu respectively (Bonmatin *et al*., 1995; Tang *et al*., 2010; Biniarz and Lukaszewicz, 2017). Common fragments of an isoform with Val/Leu/Asp/Val/Leu/Val as amino acid sequence have been found and are proposed in Fig. 1E, which is a new surfactin that, to the best of our knowledge, is not reported in the literature.

**Figure 2:**
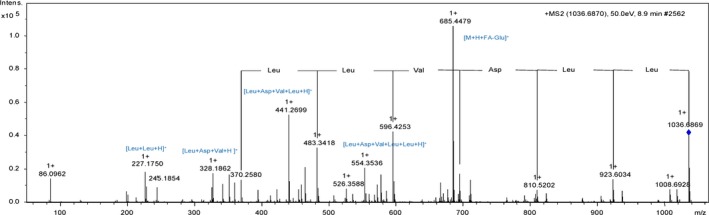
Fragmentation spectrum ESI‐(+)‐MS/MS of ion *m/z* 1036.6855 of sample *B. amyloliquefaciens* MO4B and the fragmentation proposal for isoform Surfactin A (C15).

The MS/MS spectrum of [M+H]^+^ ion with *m/z* 1036.6869 presented fragments corresponding to losses of amino acid residues Leu/Leu/Asp/Val/Leu/Leu, with *m/z* 923.6034, 810.5202, 695.4935, 596.4253, 483.3418 and 370.2570 respectively (Fig. 2). The *m/z* 370.2570 corresponded to glutamic acid residue with aliphatic fatty acid chains containing 15 carbons, indicating similarities between the proposed surfactin A (C15) produced by all five selected samples and literature surfactin (Liao *et al*., 2016). Additional fragment ions [M+H]^+^ confirmed the presence of amino acid residues sequence as *m/z* 227.1750 [Leu+Leu+H]^+^, *m/z* 328.1862 [Leu+Asp+Val+H]^+^, *m/z* 441.2699 [Leu+Asp+Val+Leu+H]^+^ and *m/z* 554.3536 [Leu+Asp+Val+Leu+Leu+H]^+^. The MS/MS spectra of [M+H]^+^ ions for all surfactins are depicted in the Supporting Information, in Figs S1–S17.

**Figure 3:**
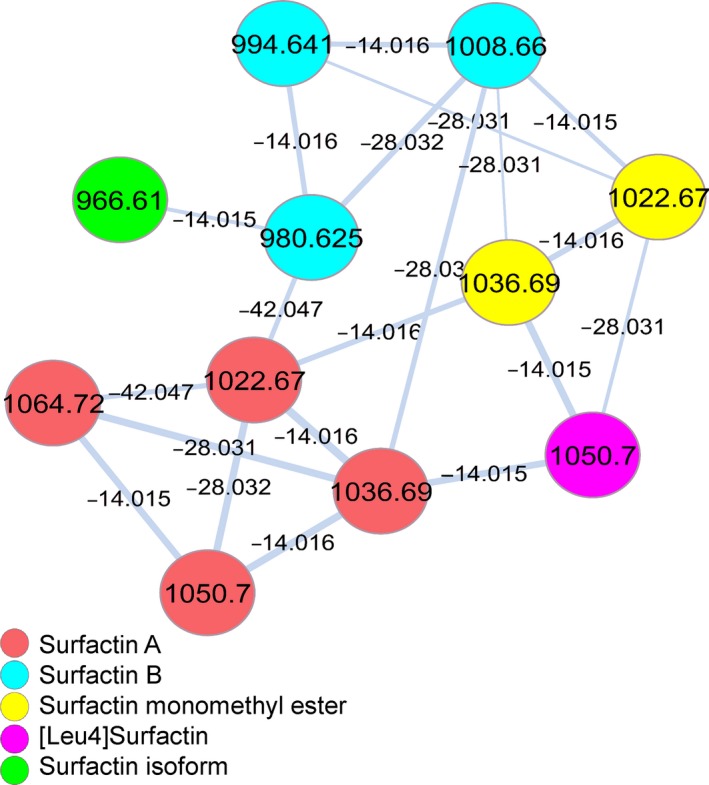
Molecular networking generated when the five selected samples of *Bacillus* were analysed via UHPLC‐MS/MS. The inlaid portions of the network were rearranged in Cytoscape for easier visualization of node connectivity.

The molecular networking analysis of the five selected extracts showed compounds with *m/z* differences of 14.015, 28.035 and 42.047, suggesting molecules with different lengths of fatty acid chains within the same isoform family (Fig. 3).

The visualization of mass differences on the chemical map leads to further investigations on the series of homologues, which were confirmed by manual interpretation of their MS/MS spectrum. Series homologue from ions [M+H]^+^ of surfactin isoforms are displayed in Table 3 for *B. amyloliquefaciens* MO4B. The same analysis was performed for all samples, and the results are depicted in the Supporting Information in Tables S1–S5.

**Table 3:** Lipopeptide composition of *Bacillus amyloliquefaciens* MO4B analysed by UHPLC‐ESI‐MS/MS

Structure	Molecular formula	[M+H]+	Observed [M+H]+	Error (ppm)
Surfactin A				
C11	C_49_H_85_N_7_O_13_	980.628361	980.6229	5.57
C12	C_50_H_87_N_7_O_13_	994.644011	994.6401	3.93
C13	C_51_H_89_N_7_O_13_	1008.65966	1008.6560	3.63
C14	C_52_H_91_N_7_O_13_	1022.67531	1022.6703	4.90
C15	C_53_H_93_N_7_O_13_	1036.69096	1036.6869	3.92
C16	C_54_H_95_N_7_O_13_	1050.70661	1050.7020	4.39
C17	C_55_H_97_N_7_O_13_	1064.72226	1064.7187	3.34
Surfactin B				
C12	C_49_H_85_N_7_O_13_	980.628361	980.6245	3.94
C13	C_50_H_87_N_7_O_13_	994.644011	994.6381	5.94
C14	C_51_H_89_N_7_O_13_	1008.65966	1008.6554	4.22
C15	C_52_H_91_N_7_O_13_	1022.67531	1022.6681	7.05
Surfactin monomethyl ester				
C13	C_52_H_91_N_7_O_13_	1022.67531	1022.6719	3.34
C14	C_53_H_93_N_7_O_13_	1036.69096	1036.6864	4.40
C16	C_55_H_97_N_7_O_13_	1064.72226	1064.7188	3.25
[Leu4] Surfactin				
C15	C_54_H_95_N_7_O_13_	1050.70661	1050.7015	4.86
Surfactin isoform				
C12	C_48_H_83_N_7_O_13_	966.612711	966.6077	5.18
C15	C_51_H_89_N_7_O_13_	1008.65966	1008.6553	4.32

### Critical micelle concentration

**Figure 4:**
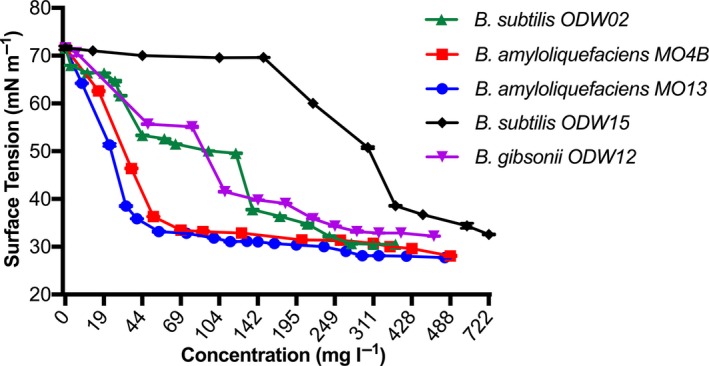
Surface tension values (mN/m) versus biosurfactant concentration (mg l^−1^) obtained with the cell‐free crude biosurfactant produced by bacterial isolates *B. subtilis* ODW15, *B. gibsonii* ODW12, *B. subtilis* ODW02, *B. amyloliquefaciens* MO4B and *B. amyloliquefaciens* MO13, dissolved in deionized water. Results represent the average of three independent measurements ± standard deviation.

The CMC is the minimum concentration of a tensoactive compound necessary to reduce the surface tension to the maximum extent. The CMC is a characteristic property of each tensoactive compound, and it is commonly used to define its efficiency (Ferhat *et al*., 2011). Measurement of the CMC was performed for the five most promising isolates, *B. subtilis* ODW15, *B. gibsonii* ODW12, *B. subtilis* ODW02, *B. amyloliquefaciens* MO4B and *B. amyloliquefaciens* MO13, using the cell‐free crude biosurfactant. The results are shown in Fig. 4.

According to Zhang and Miller (1992), the concentration needed to reduce the water surface tension from 71.2 mN m^−1^ to values below 40 mN m^−1^ is typically between 1 and 200 mg l^−1^. As can be seen in Fig. 4, in our samples four out of the five isolates have been able to reduce the water surface tension below 40 mN m^−1^ in CMC below 200 mg l^−1^. The only exception is *B. subtilis* ODW15, which needed higher concentrations (389 mg l^−1^) to reduce the water surface tension. The most efficient isolate, *B. amyloliquefaciens* MO13, displayed a CMC of 36 mg l^−1^ and was able to reduce the water surface tension to 27 mN m^−1^. The efficiency of the biosurfactant produced by *B. amyloliquefaciens* MO13 is noteworthy and is comparable with the surfactant produced by *B. subtilis* recovered from cassava waste which displayed a surface tension of 26.6 mN m^−1^ and a CMC of 33 mg l^−1^ (Nitschke and Pastore, 2006).

### Study of the stability of the biosurfactant

**Figure 5:**
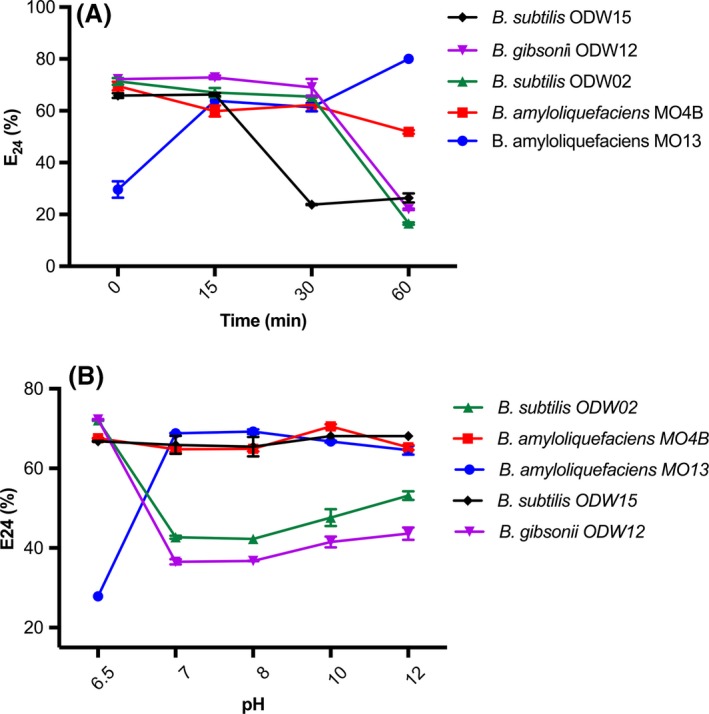
(A) Emulsification activity (%) versus time (minutes), obtained after heating the samples at 100°C for 15, 30 and 60 min. (B) Emulsification activity (%) versus pH. Results represent the average of three independent measurements ± standard deviation.

The chemical stability of the biosurfactants produced was evaluated towards a set of different conditions, such as autoclaving, temperature, salt concentration and pH (Fig. 5). The biosurfactants' stability in terms of surface tension was not affected following the variation of any of the specific parameters. On the other hand, emulsification activity was affected in response to changes in temperature and pH.

For example, autoclaving the samples did not result in any change on the surface tension or emulsification activity. On the other hand, when the crude biosurfactant was heated at 100°C for 15, 30 and 60 min a more pronounced difference in the behaviour was observed for all samples (Fig. 5A). The emulsification ability of *B. subtilis* ODW15, *B. gibsonii* ODW12, *B. subtilis* ODW02 was reduced, while *B. amyloliquefaciens* MO13 increased significantly after heating. On the other hand, *B. amyloliquefaciens* MO4B has shown a higher thermal stability and a smaller decrease in the emulsification ability was observed after 1 h of heating at 100°C. For all, the surface tension resulted unchanged, thereby attesting for the thermal stability of the compounds produced (Fig. 5A). Addition of increasing amounts of NaCl (from 0.5% to 35%) did not affect the tensoactive properties.

Finally, the influence of the pH was also evaluated (Fig. 5B). We have evaluated the stability of the biosurfactant in either acidic or basic conditions using pH values varying from 2 to 12. The initial pH values for all samples were 6.5. In any of the pH values studied, we did not observe changes in the ability of the surfactant to reduce the water surface tension. However, when the pH was lowered to 4 and 2, the emulsification activity has ceased, likely due to partial precipitation of the biosurfactant, because of the presence of the carboxyl groups at the surfactin structure. In strongly acidic conditions, the carboxylate will be protonated and the surfactin becomes less soluble in water, losing its emulsification ability. This feature was already reported in the literature (Nitschke and Pastore, 2006) and allowed the recovery of surfactins by acidic precipitation. On the other hand, under neutral or basic conditions (pH values of 7, 8, 10 and 12) a strong decrease in the emulsification activity was observed for *B. gibsonii* ODW12 and *B. subtilis* ODW02. Conversely, *B. amyloliquefaciens* MO4B has shown stable values for E_24_ and *B. subtilis* ODW15 did not display any significative difference for E_24_ values. A significant increase in E_24_ was observed again for *B. amyloliquefaciens* MO13. In this case, the more basic the medium, the more ionized the carboxyl groups of the surfactins and, therefore, the compounds become more water‐soluble. As a consequence, the ability to stabilize the emulsions also increases (Long *et al*., 2017). This different behaviour depending on the acidity/basicity of the medium renders to surfactins the property of a pH‐responsive tensoactive agent.

In summary, we have reported the screening of biosurfactant‐producing bacteria isolated from areas contaminated with crude oil or its byproducts. Five of two hundred isolates furnished significant decrease in surface tension below 30 mN m^−1^. Worth pointing out is that *B. amyloliquefaciens* MO4B has shown to be a very promising biosurfactant producer, particularly, due to its ability to efficiently reduce the surface tension and emulsification ability that remained stable even when the samples were submitted to pH changes or exposed to high temperatures. The biosurfactants produced were fully chemically characterized via UPLC‐HRMS, indicating the production of surfactin homologues, including a new member of this family is reported for the first time in the literature.

## Experimental procedures

### Microorganism isolation

The microorganisms used in this study were isolated from oil‐contaminated soil samples obtained from different Brazilian terrestrial sites: (i) isolates of petroleum‐contaminated soil (1:20 m/m) and oil‐contaminated soil collected at gas stations in the city of Porto Alegre (RS) from the inventory of the Laboratory of Fungi of Medical and Biotechnological Importance from Universidade Federal do Rio Grande do Sul (UFRGS), Porto Alegre, RS, Brazil; (ii) oil‐drilling waste (ODW) samples obtained from a petroleum production batch at the northeastern coast of the city of Mossoró, Rio Grande do Norte, Brazil.

The cultures were grown in Erlenmeyer flasks (250 ml) containing 50 ml Luria‐Bertani (LB) (Thermo Fisher Scientific, Waltham, Massachusetts, USA.) or 50 ml YPD (glucose 20 g l^−1^, peptone 20 g l^−1^, yeast extract 10 g l^−1^). The flasks with both samples were kept in agitation at 200 rpm for 7 days at 30°C. After this period, aliquots (100 μl) of this mixture were subjected to successive decimal dilutions and were then plated on LB agar and YPD. The plates were incubated for 5 days at 30°C. Microorganisms with morphological differences were transferred to new media to obtain pure colonies. The microorganisms isolated were kept refrigerated at 4°C.

### Screening for biosurfactant production

To assess the biosurfactant‐producing ability of the microorganisms, the isolates were inoculated in YPD liquid medium on a rotary shaker at 180 rpm at 30°C for 24 h. The isolates were then subjected to liquid fermentation in minimal salt medium (MSM) (Na_2_HPO_4_ (6 g l^−1^), KH_2_PO_4_ (3 g l^−1^), NaCl (0.5 g l^−1^), NH_4_Cl (1 g l^−1^), MgSO_4_.7H_2_O, (0.24 g l^−1^), using glucose (5%) as the only carbon source. The samples were incubated on a rotary shaker at 180 rpm for 5 days at 30°C. Following incubation, cultures were centrifuged at 9000 rpm for 10 min, and the cell‐free supernatant was used to test biosurfactant activity (Belgacem *et al*., 2015). *Pseudomonas aeruginosa* ATCC 27853, which has previously been described as a biosurfactant producer, was used as the positive control (Sakthipriya *et al*., 2015).

#### Drop‐collapse test

Drop‐collapse tests were carried out according to the method described by Jain *et al*. (1991) and modified by Bodour and Maier (1998). The tests were carried out on a clear flat surface. About 1.8 μl of oil (SINGER^™^, São Paulo, Brazil.) and 20 μl of supernatant were added to the oil surface. The shape of the droplet was inspected after 1 min. If the drop remained beaded, the assay was scored negative and if the drop collapsed, the result was considered positive.

#### Oil‐spreading assay

Fifty milliliter of distilled water was brought into a large Petri dish (15 cm diameter) followed by the addition of 200 μl of oil to the water surface to form a thin oil layer. About 20 μl of the supernatant was then carefully added to the center of the oil layer, and the diameter of the clearing zone was measured. If biosurfactants are present, the oil will be displaced, resulting in an oil‐free clearing zone where the diameter correlated with the surfactant activity (Pornsunthorntawee *et al*., 2008).

The combined results of these two assays were used to select the most promising microorganisms for further evaluation of the emulsification capacity and surface tension.

#### Emulsification activity (E_24_)

The emulsification activity (E_24_) was measured using the method described by Cooper and Goldenberg (1987). The test was performed by mixing 2 ml of kerosene with an equal volume of supernatant (vortexed for 2 min) and left to stand for 24 h. Emulsions formed by each isolate were compared with those formed by negative control. The emulsification activity was calculated as the ratio of the height of the emulsion layer (cm) and the total height of liquid in the tube (cm), multiplied by 100.

#### Surface tension measurement

The surface tension was measured using a digital tensiometer (Gibertini, Milan, Italy). Fifteen milliliter of the supernatant of the microorganism cultures was used. Distillated water and ethanol (96%) were used as standards. The measurements were made by immersing a coverslip underneath the surface of the supernatant (ca 1 mm), which was then slowly pulled out, the maximum force was then measured and taken.

### Molecular identification of microorganisms

The genomic DNA of each culture was extracted according to Sambrook *et al*. (2001). A fragment of the V3 region of the bacterial 16S rRNA gene was amplified from microbial DNA using the primers 27F (5′ AGA GTT TGA TCM TGG CTC AG 3′) and 1492R (5′ GGT TAC CTT GTT ACG ACT T 3′). Amplification was performed in a reaction containing 20 ng μl^−1^ of DNA template, 1 U Platinum Taq DNA Polymerase (Invitrogen), 1X Taq buffer, 1.5 mM MgCl_2_, 0.2 mM dNTP and 10 pmol of each primer, resulting in a final volume of 50 μl. The amplifications were carried out as follows: one cycle at 94°C for 5 min, followed by 30 cycles at 94°C for 1 min, 60°C for 0.3 min, 72°C for 2 min and a final extension step at 72°C for 6 min.

The PCR products were analysed with 1% agarose gel electrophoresis. The amplicons obtained were purified from Kit PureLink™ Quick Gel Extraction (Invitrogen, Carlsbad, California, USA), according to the manufacturer's instructions. Approximately, 200 ng of genomic DNA was used for sequencing on a BI‐Prism 3500 Genetic Analyzer Platform (Applied Biosystems). Nucleotide sequence similarity searches were conducted using the GenBank nucleotide collection BLAST searches.

### Biosurfactant identification

#### Extraction of the biosurfactant

Liquid fermentation was performed in 500 ml Erlenmeyer flaks using 150 ml MSM supplemented with 5% glucose. Following incubation at 180 rpm for 5 days at 30°C, the cells were removed by centrifugation at 9000 rpm for 10 min. The pH of the supernatant was adjusted to 2 using concentrated HCl, and the supernatant was left standing for 24 h at 4°C and it was then extracted three times using chloroform/methanol (2:1 v/v) in a separatory funnel for the extraction of organic compounds. The organic layer was separated and dried with anhydrous sodium sulphate to remove water traces, and it was then evaporated under reduced pressure in a rotary evaporator at 45°C, yielding a viscous, brown oil (Belgacem *et al*., 2015).

#### Preliminary identification of the biosurfactants by TLC

To preliminary determine the type of biosurfactant, a portion of the crude extract was separated on silica gel TLC plates (F254, 0.2 mm thickness) using ethyl acetate as running solvent agent. For the detection of lipopeptides, the plates were air‐dried, sprayed with 0.5% ninhydrin (VWR), once again air‐dried and then heated for 15 min at 110°C for colour development. Anisaldehyde reagent (100:2:1 (v/v/v) acetic acid:sulphuric acid:*p*‐anisaldehyde (Sigma)) was used for carbohydrate detection. Anthrone reagent (63 ml of sulphuric acid, 25 ml of water and 0.125 g of anthrone (Sigma)) was used for sugar detection and the colour was developed by heating plates at 110°C for 10 min. Lipid components were detected after placing the plates in a closed jar saturated with iodine vapour. Once visible, the retention factor (*Rf*) of each spot was determined by dividing the distance travelled by the product by the total distance travelled by the solvent (Belgacem *et al*., 2015).

#### High‐resolution mass spectrometry analysis

Crude extracts were analysed by UHPLC (Shimadzu, Nexera X2) coupled with HRMS (Impact II, Bruker Daltonics Corporation, USA) equipped with an electrospray ionization source. The capillary voltage was operated in positive ionization mode, set at 4500 V, and with an endplate offset potential of −500 V. The dry gas parameters were set to 8 l min^−1^ at 200°C with a nebulization gas pressure of 4 bar. Data were collected from *m/z* 50–1600 with an acquisition rate of five spectrums per second, and the ions of interest were selected by auto MS/MS scan fragmentation. Chromatographic separation was performed with a gradient mixture of solvents A H_2_O (0.1% formic acid v/v) and acetonitrile B (0.1% formic acid v/v) using a C18 column (ACQUITY UPLC BEH C18 1.7 μm 2.1 × 100 mm; Waters, USA). The gradient is described in Table 4.

**Table 4:** Gradient programme used for the separation of the crude extracts

Time (min)	% Solvent B	Flow (ml min^−1^)
1.0	60	0.200
3.0	70	0.200
20.0	98	0.200
21.0	2	0.200
22.0	60	0.200
25.0	60	0.200
25.0	stop	–

#### MS/MS data processing – molecular networking

Raw data from UHPLC‐MS/MS analysis were converted to mzXML format with Bruker's Data Analysis software and uploaded to the global natural product social (GNPS) molecular networking tools. In GNPS (Wang *et al*., 2016), the data were subjected to molecular networking using the online workflow at GNPS. The data were clustered with MS‐Cluster with a parent mass tolerance of 1.0 Da and a MS/MS fragment ion tolerance of 0.5 Da to create consensus spectra. A network was then created where edges parameters were cosine score above 0.65 and more than four matched peaks. The data were filtered by removing all MS/MS peaks within ± 17 Da of the precursor *m/z*. The network was then searched against GNPS's spectral libraries, and all matches between network spectra and library spectra had requisite to present a score above 0.65 and at least four matched peaks. The network analysis was exported from GNPS and analysed in Cytoscape (Shannon *et al*., 2003).

### Critical micelle concentration

At the CMC, an increasing concentration of the surfactant will lead to a sudden change in the decreasing rate of surface tension. Different concentrations of the biosurfactants' crude extracts were prepared in distilled water, and the changes in surface tension values were measured at 25°C. The CMC was determined from the inflection point of surface tension versus biosurfactant concentration (Nitschke and Pastore, 2006).

### Studies of the stability of the biosurfactant

The biosurfactants' stability was evaluated using the cell‐free supernatant, according to the methodology adapted from Abdel‐Mawgoud *et al*. (2008). The effect of temperature on the biosurfactants' stability was evaluated by heating the samples in a water bath for 15, 30 and 60 min at 100°C; the samples were then allowed to cool to room temperature. After the designated times, the surface tension and emulsification activity were measured for each sample to check for any possible changes.

To measure the biosurfactants' salinity stability, NaCl was added to the supernatant until final saline concentration of 2.5%, 5%, 10%, 20% and 35% was reached. After the addition of NaCl, the solutions were stirred to complete dissolution. The resulting solutions were then incubated for 30 min at 30°C before being allowed to reach room temperature. The surface tension of each sample was then measured to check for any possible changes.

To measure the effect of pressure, the supernatant was placed in an autoclave at 1 atm for 15 min at 121°C. Analysis of pH stability was performed by adjusting the pH of the supernatant to 2, 4, 7, 8, 10 and 12. The surface tension and emulsification activity were then measured to check for any possible changes.

### Statistical analysis

The data are presented in terms of arithmetic averages of at least three replicates, and the error bars indicate the standard deviations. The analyses were carried out using ANOVA, followed by Tukey test with a confidence level of 95%.

## Conflict of Interest

The authors declare that there are no conflict of interests

## Supporting information


**Fig. S1.** Surfactin A (C11).
**Fig. S2.** Surfactin A (C12).
**Fig. S3.** Surfactin A (C13).
**Fig. S4.** Surfactin A (C14).
**Fig. S5.** Surfactin A (C15).
**Fig. S6.** Surfactin A (C16).
**Fig. S7.** Surfactin A (C17).
**Fig. S8.** Surfactin B (C12).
**Fig. S9.** Surfactin B (C13).
**Fig. S10.** Surfactin B (C14).
**Fig. S11.** Surfactin B (C15).
**Fig. S12.** Surfactin monomethyl ester (C13).
**Fig. S13.** Surfactin monomethyl ester (C14).
**Fig. S14.** Surfactin monomethyl ester (C16).
**Fig. S15.** [Leu4] Surfactin (C15).
**Fig. S16.** Surfactin isoform (C12).
**Fig. S17.** Surfactin isoform (C15).
**Table S1.** Lipopeptide composition of *B. amyloliquefaciens* MO13 analyzed by UHPLC‐ESI‐MS/MS.
**Table S2.** Lipopeptide composition of *B. amyloliquefaciens* MO4B analyzed by UHPLC‐ESI‐MS/MS.
**Table S3.** Lipopeptide composition of *B. subtilis* ODW 02 analyzed by UHPLC‐ESI‐MS/MS.
**Table S4.** Lipopeptide composition *B. gibsonii* ODW12 analyzed by UHPLC‐ESI‐MS/MS.
**Table S5.** Lipopeptide composition *B. subtilis* ODW15 analyzed by UHPLC‐ESI‐MS/MS.
