# Supplementary material for: Identification and ultra‐high‐performance liquid chromatography coupled with high‐resolution mass spectrometry characterization of biosurfactants, including a new surfactin, isolated from oil‐contaminated environments
Source: Microb Biotechnol. 2018 May 14;11(4):759–69. doi: 10.1111/1751-7915.13276 (PMC6011949; doi:10.1111/1751-7915.13276)
Supplement: Supplementary file 1 — Fig. S1. Surfactin A (C11). Fig. S2. Surfactin A (C12). Fig. S3. Surfactin A (C13). Fig. S4. Surfactin A (C14). Fig. S5. Surfactin A (C15). Fig. S6. Surfactin A (C16). Fig. S7. Surfactin A (C17). Fig. S8. Surfactin B (C12). Fig. S9. Surfactin B (C13). Fig. S10. Surfactin B (C14). Fig. S11. Surfactin B (C15). Fig. S12. Surfactin monomethyl ester (C13). Fig. S13. Surfactin monomethyl ester (C14). Fig. S14. Surfactin monomethyl ester (C16). Fig. S15. [Leu4] Surfactin (C15). Fig. S16. Surfactin isoform (C12). Fig. S17. Surfactin isoform (C15). Table S1. Lipopeptide composition of B. amyloliquefaciens MO13 analyzed by UHPLC‐ESI‐MS/MS. Table S2. Lipopeptide composition of B. amyloliquefaciens MO4B analyzed by UHPLC‐ESI‐MS/MS. Table S3. Lipopeptide composition of B. subtilis ODW 02 analyzed by UHPLC‐ESI‐MS/MS. Table S4. Lipopeptide composition B. gibsonii ODW12 analyzed by UHPLC‐ESI‐MS/MS. Table S5. Lipopeptide composition B. subtilis ODW15 analyzed by UHPLC‐ESI‐MS/MS. [file MBT2-11-759-s001.pdf]

Supporting Information

Identification and UHPLC-HRMS Characterization of Biosurfactants, Including a New Surfactin, Isolated from Oil-Contaminated Environments

Glaci V. Moro,[a] Rafaela T. R. Almeida,[b] Amanda P. Napp,[a] Carla Porto,[b] Eduardo J. Pilau,[b] Diogo S. Lüdtke,[c] Angélica V. Moro,[c,*] Marilene H. Vainstein[a,*]

[a] *Centro de Biotecnologia, Universidade Federal do Rio Grande do Sul (UFRGS), Av. Bento Gonçalves 9500, 91501-970, Porto Alegre, RS, Brazil*
[b] *Departamento de Química, Universidade Estadual de Maringá (UEM), 87020-900, Maringá-PR, Brazil*
[c] *Institute of Chemistry, Universidade Federal do Rio Grande do Sul (UFRGS), Av. Bento Gonçalves 9500, 91501-970, Porto Alegre, RS, Brazil*

B. amyloliquefaciens MO4B

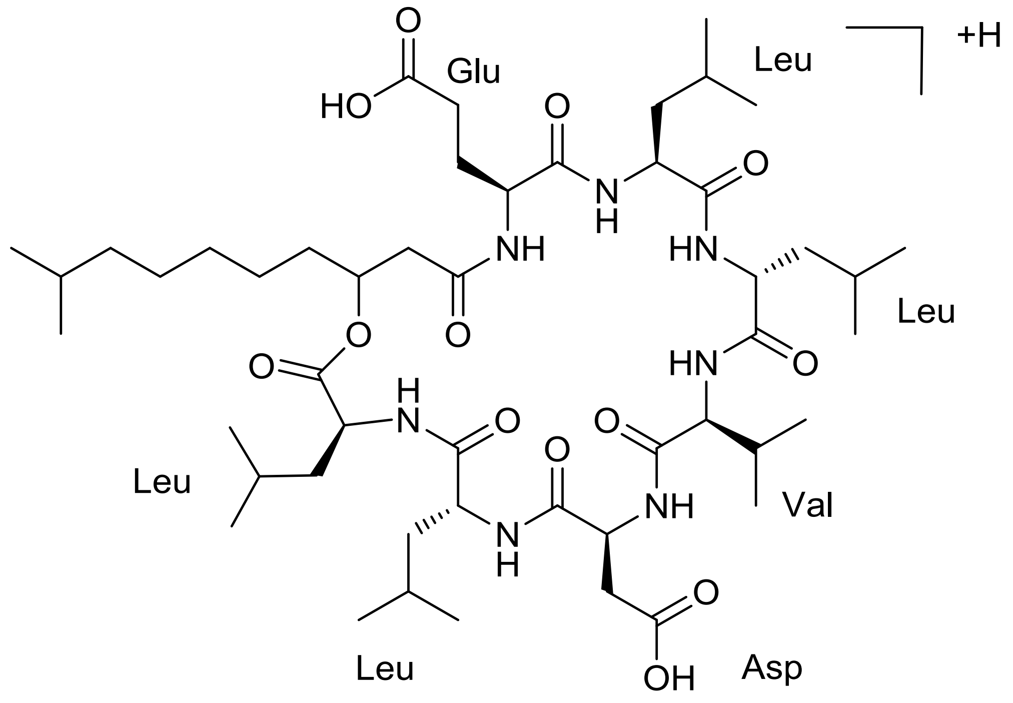

Surfactin A (C11)
Exact Mass: 980.6284

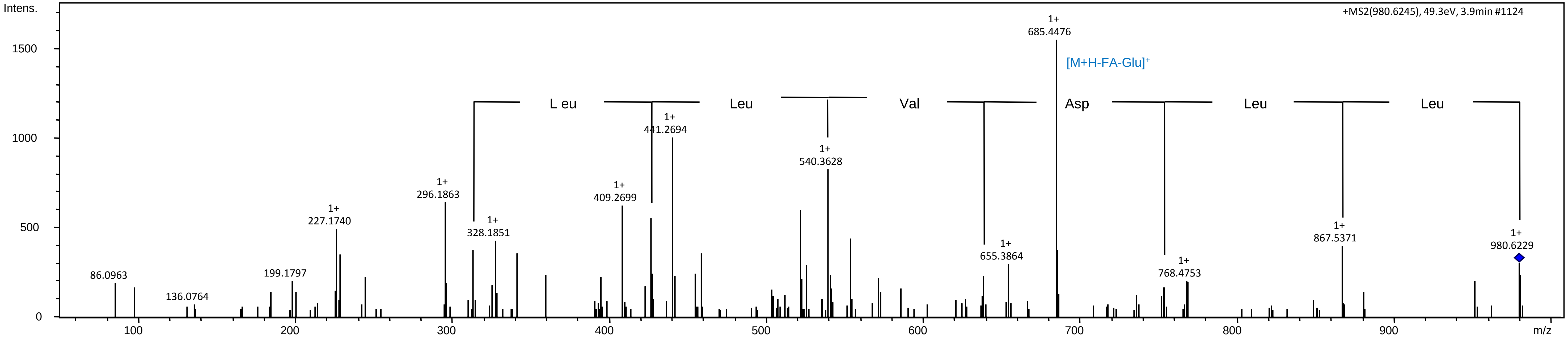

Figure S1. Surfactin A (C11). Tandem mass spectrometry (UHPLC-HRMS/MS) was used to identify the [M+H] ion at 5.57 ppm mass error. Identified fragments loss are depicted in the structure insert.

B. amyloliquefaciens MO4B

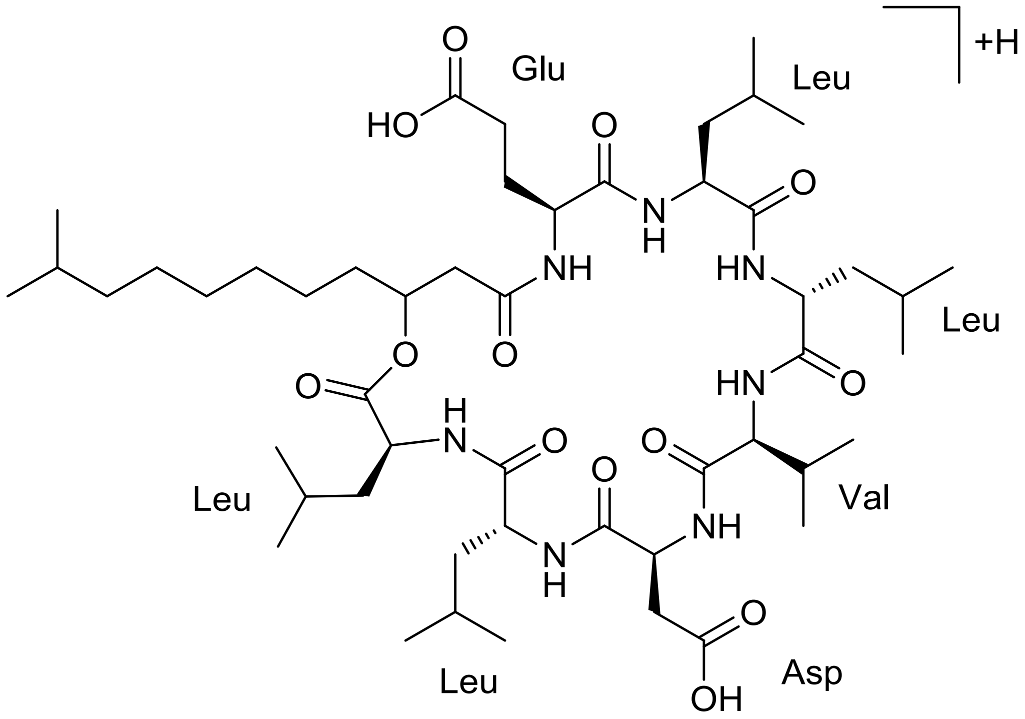

Surfactin A (C12)

Exact Mass: 994.6440

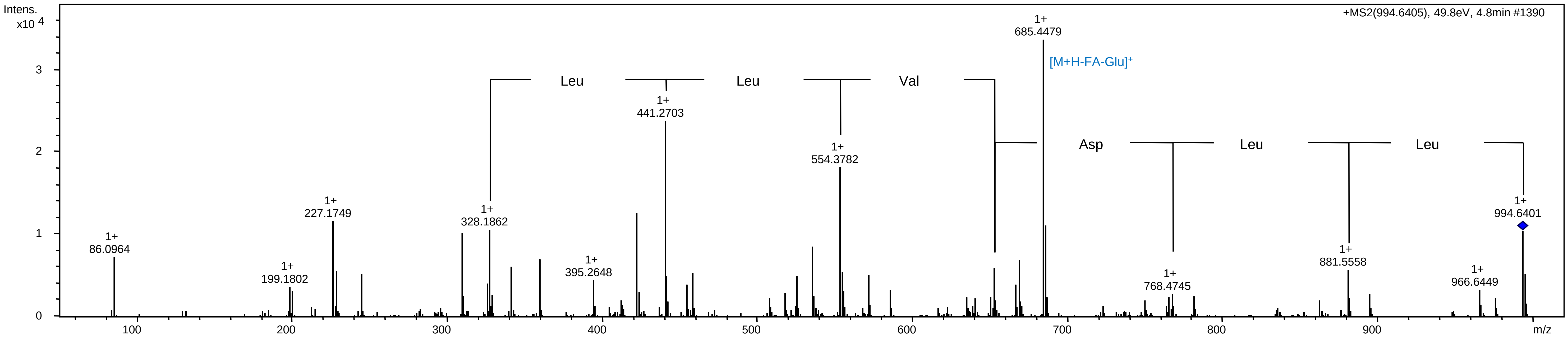

Figure S2. Surfactin A (C12). Tandem mass spectrometry (UHPLC-HRMS/MS) was used to identify the [M+H] ion at 3.93 ppm mass error. Identified fragments loss are depicted in the structure insert.

B. amyloliquefaciens MO4B

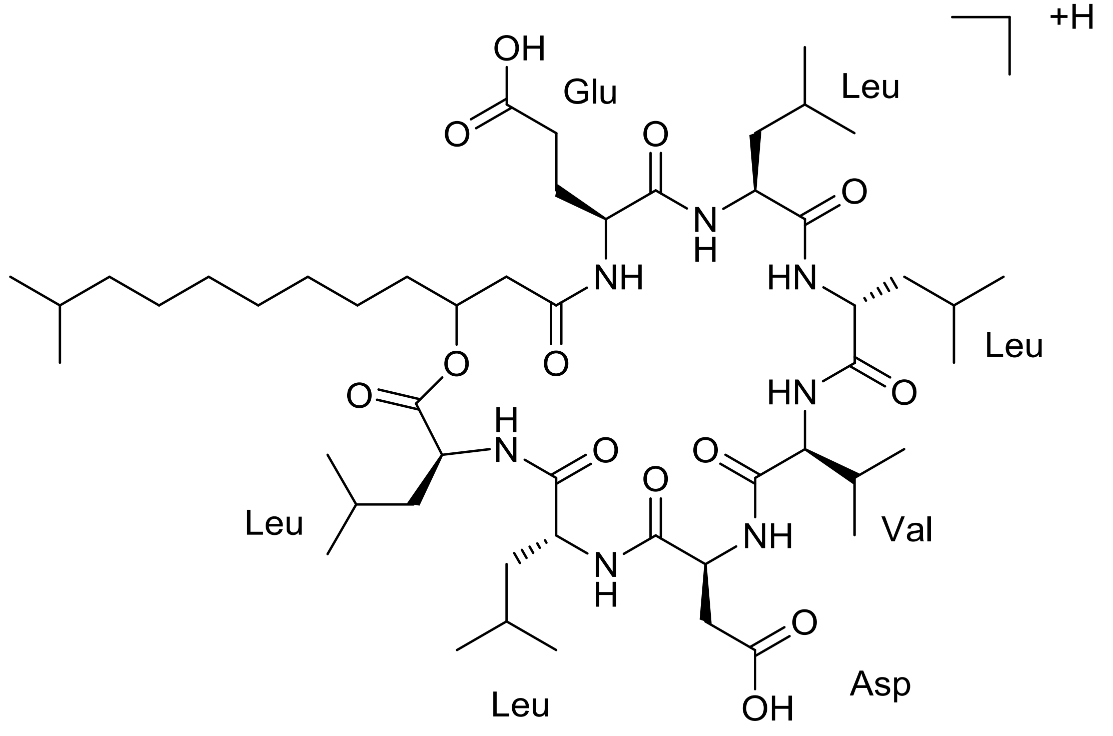

Surfactin A (C13)

Exact Mass: 1008.6597

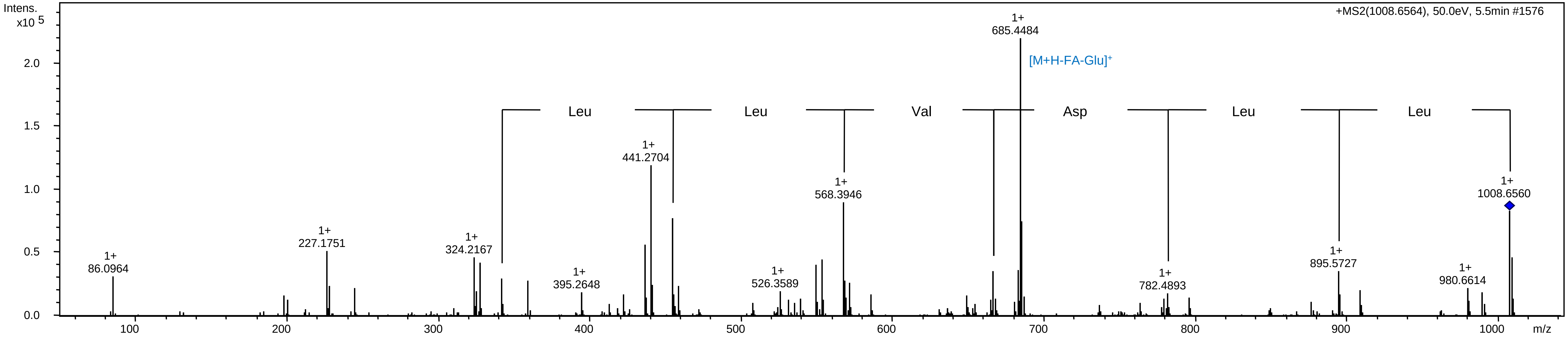

Figure S3. Surfactin A (C13). Tandem mass spectrometry (UHPLC-HRMS/MS) was used to identify the [M+H] ion at 3.63 ppm mass error. Identified fragments loss are depicted in the structure insert.

B. amyloliquefaciens MO4B

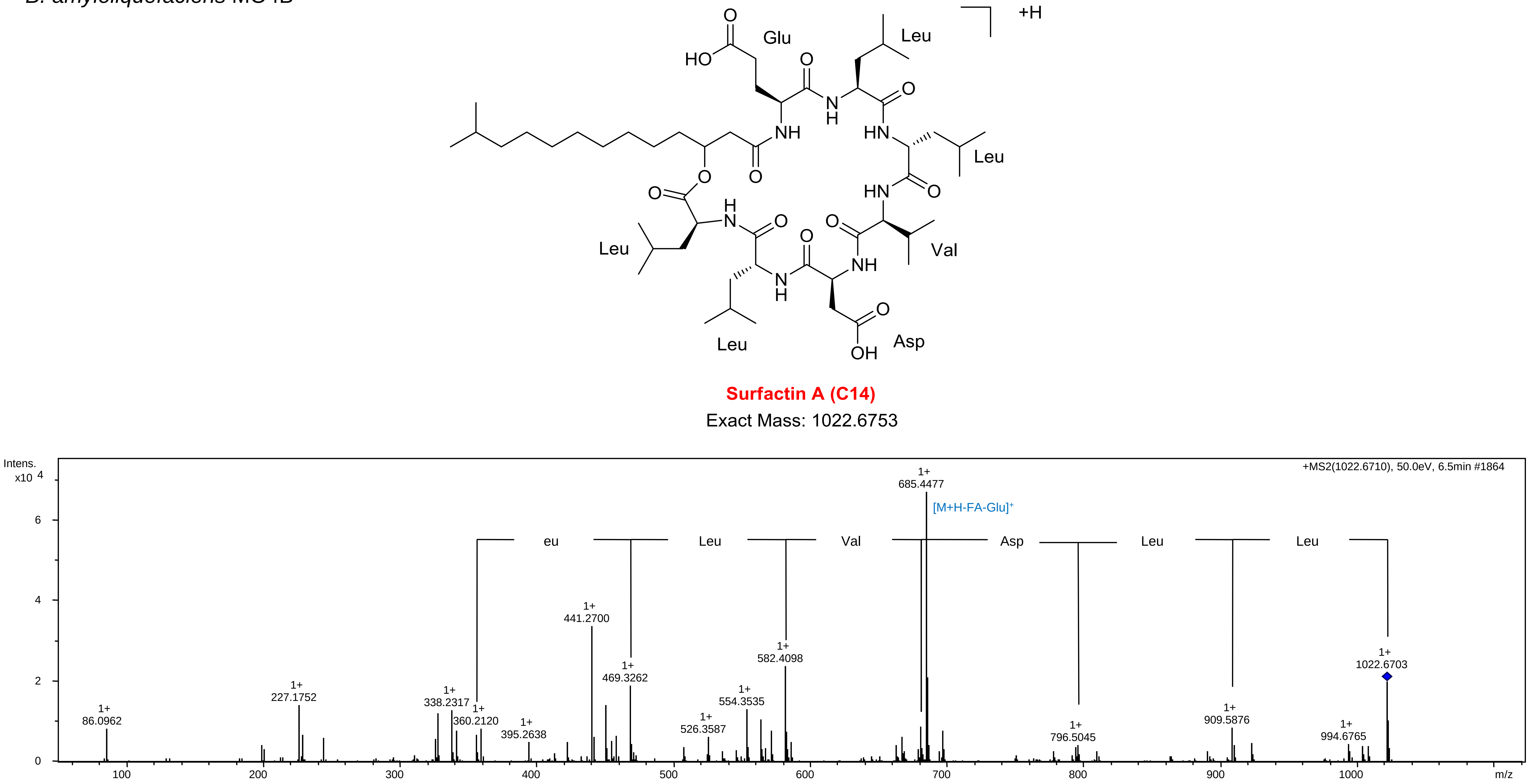

Figure S4. Surfactin A (C14). Tandem mass spectrometry (UHPLC-HRMS/MS) was used to identify the [M+H] ion at 4.90 ppm mass error. Identified fragments loss are depicted in the structure insert.

B. amyloliquefaciens MO4B

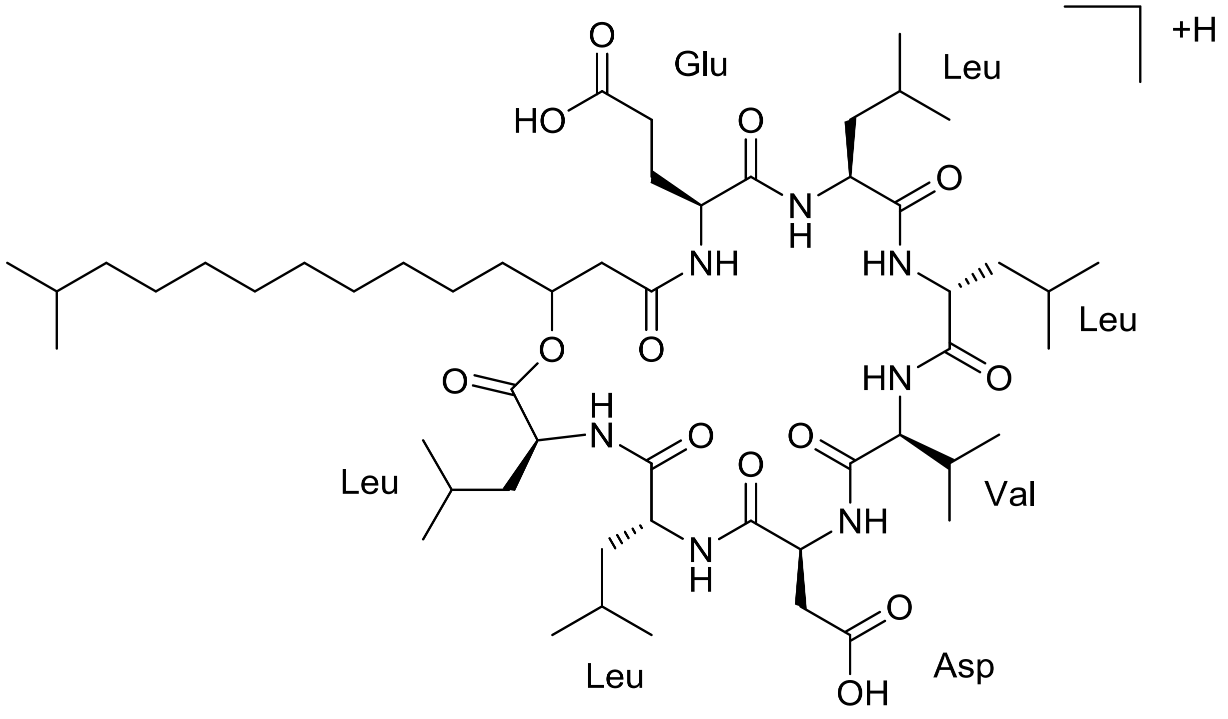

Surfactin A (C15)

Exact Mass: 1036.6910

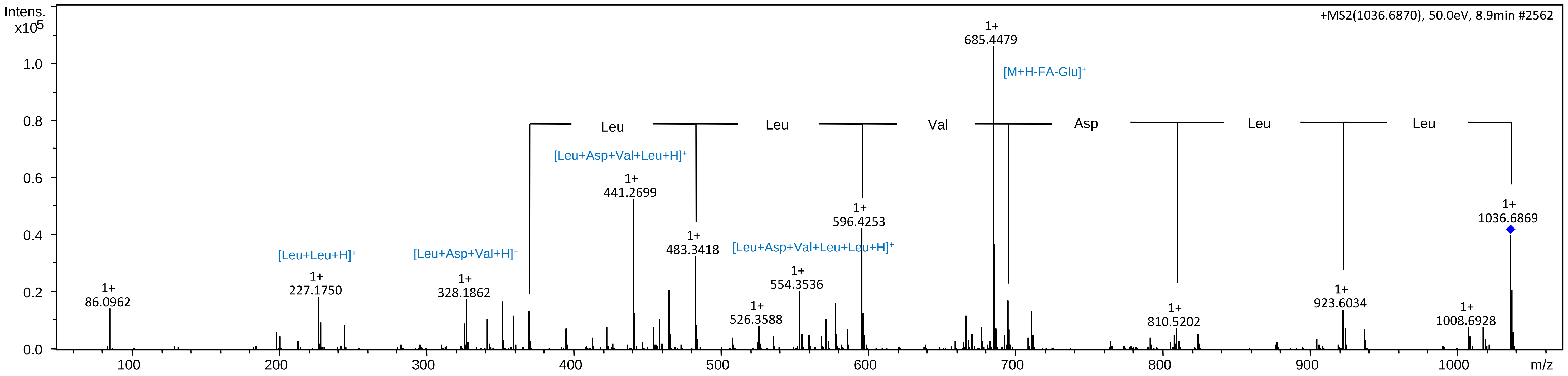

Figure S5. Surfactin A (C15). Tandem mass spectrometry (UHPLC-HRMS/MS) was used to identify the [M+H] ion at 3.92 ppm mass error. Identified fragments loss are depicted in the structure insert.

B. amyloliquefaciens MO4B

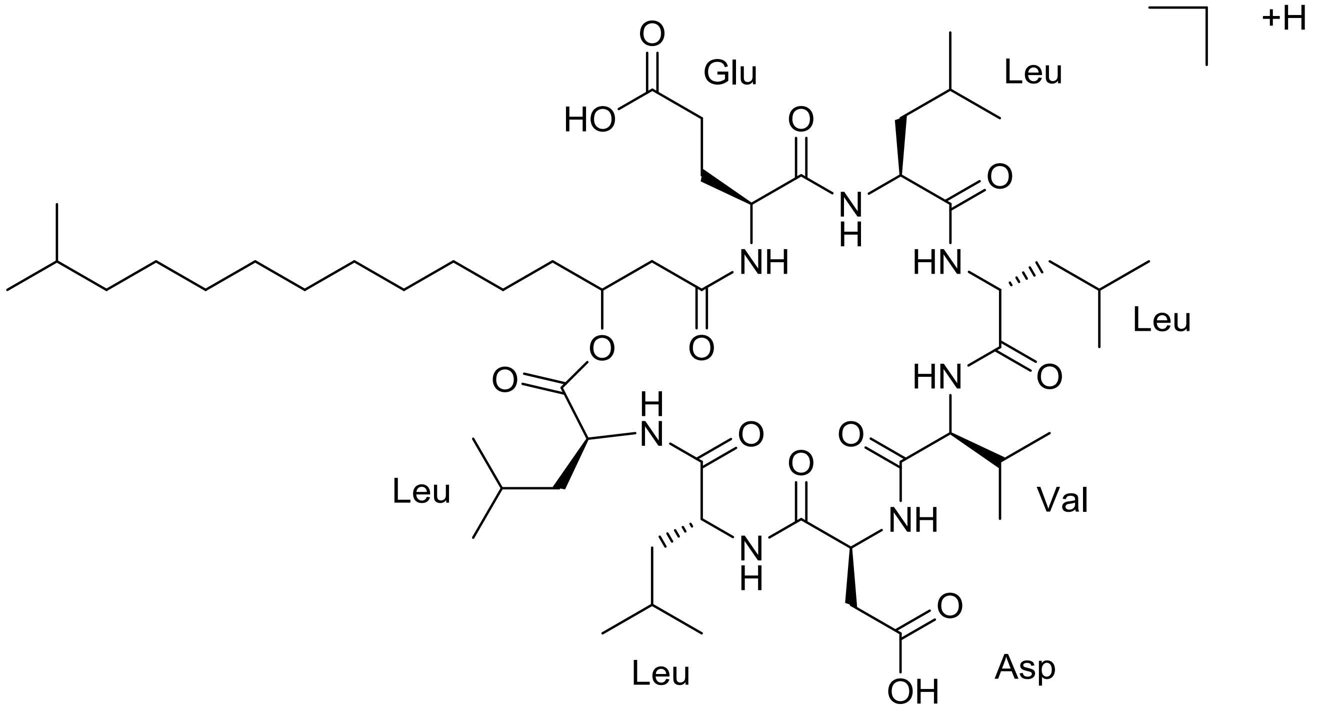

Surfactin A (C16)

Exact Mass: 1050.7066

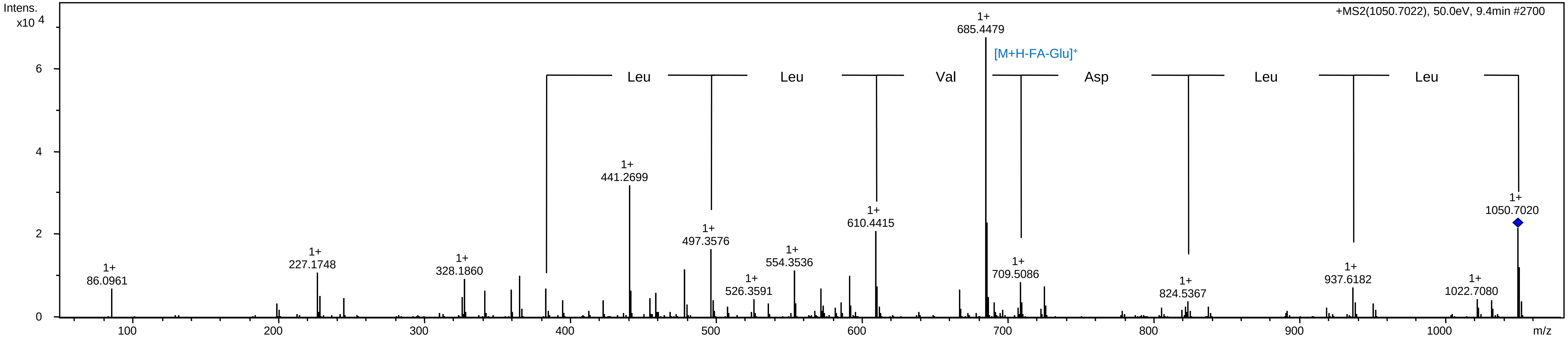

Figure S6. Surfactin A (C16). Tandem mass spectrometry (UHPLC-HRMS/MS) was used to identify the [M+H] ion at 4.39 ppm mass error. Identified fragments loss are depicted in the structure insert.

B. amyloliquefaciens MO4B

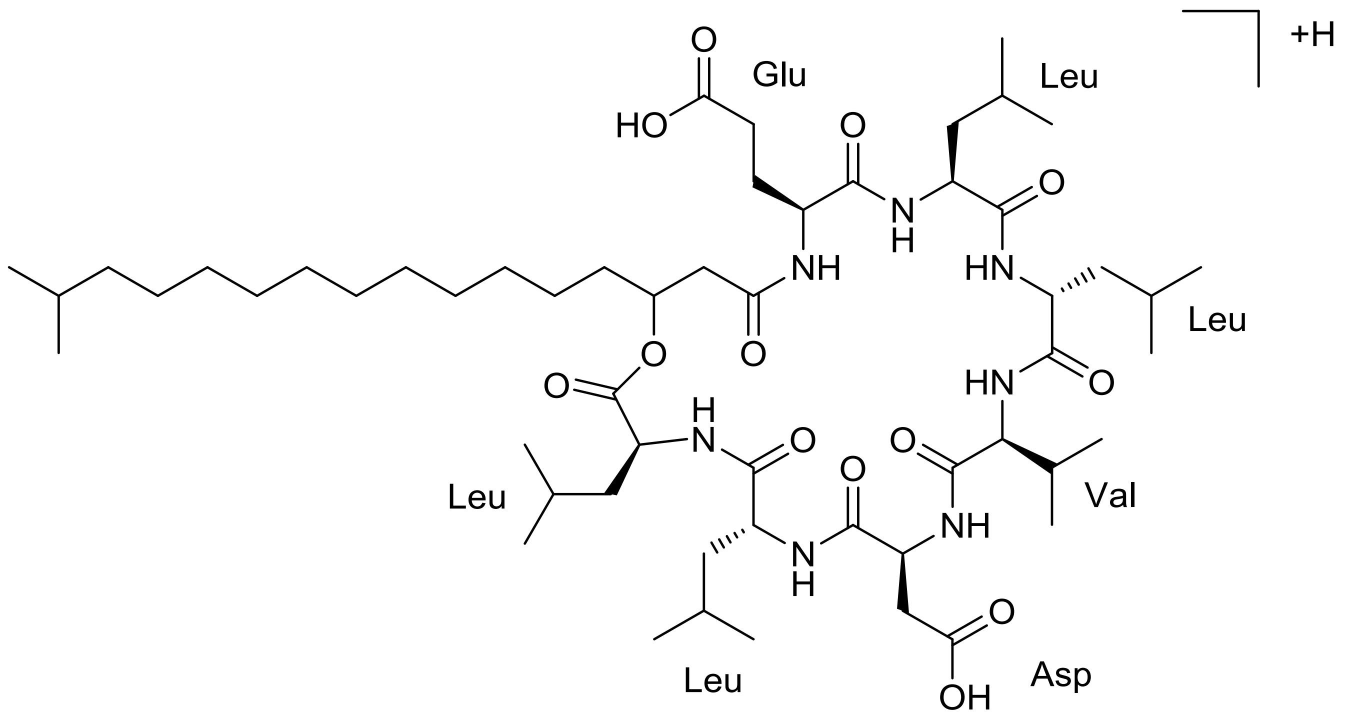

Surfactin A (C17)

Exact Mass: 1064.7223

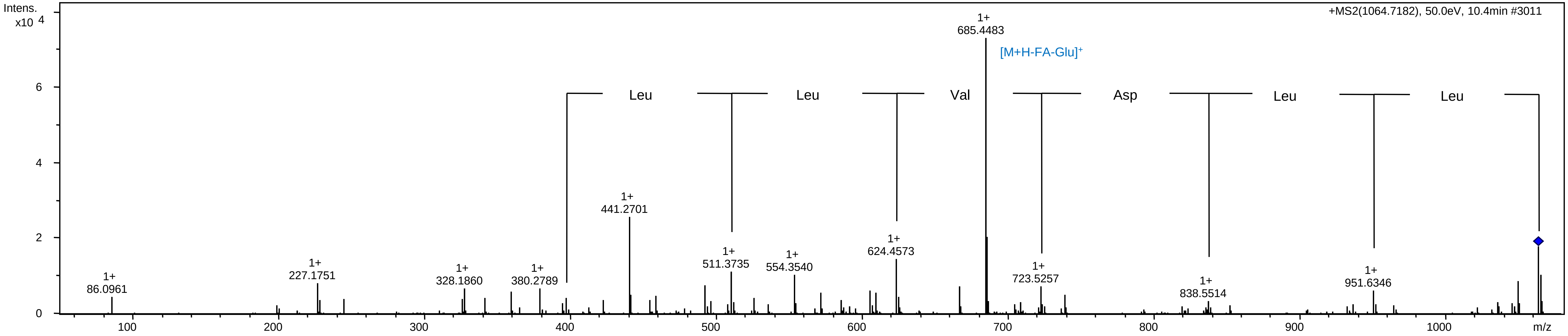

Figure S7. Surfactin A (C17). Tandem mass spectrometry (UHPLC-HRMS/MS) was used to identify the [M+H] ion at 3.34 ppm mass error. Identified fragments loss are depicted in the structure insert.

B. amyloliquefaciens MO4B

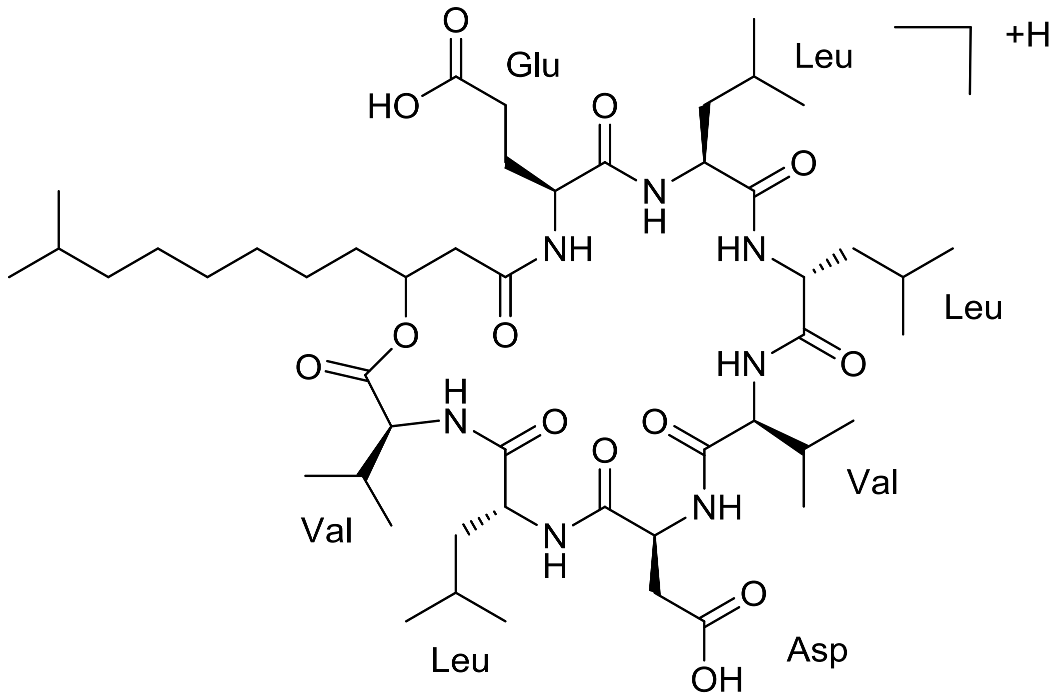

Surfactin B (C12)

Exact Mass: 980.6284

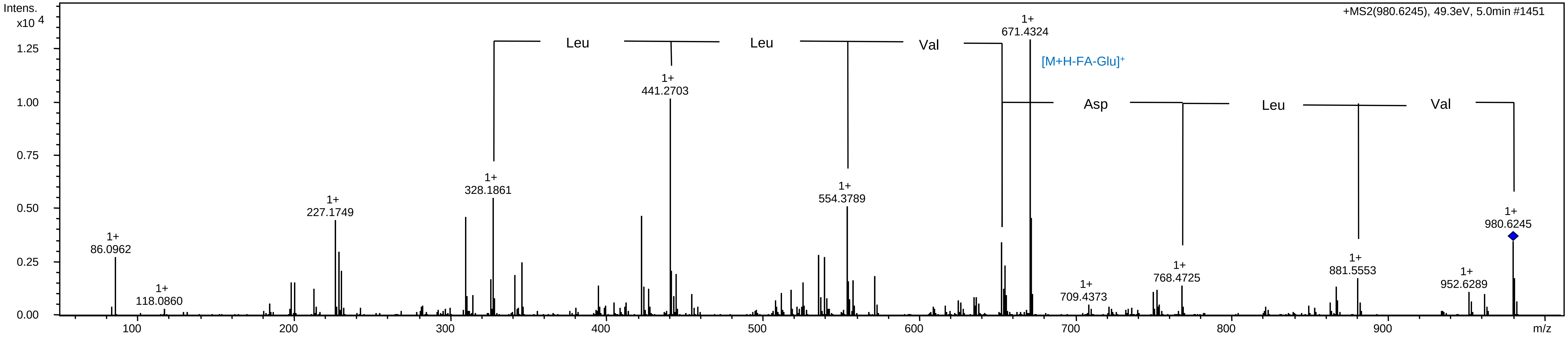

Figure S8. Surfactin B (C12). Tandem mass spectrometry (UHPLC-HRMS/MS) was used to identify the [M+H] ion at 3.94 ppm mass error. Identified fragments loss are depicted in the structure insert.

B. amyloliquefaciens MO4B

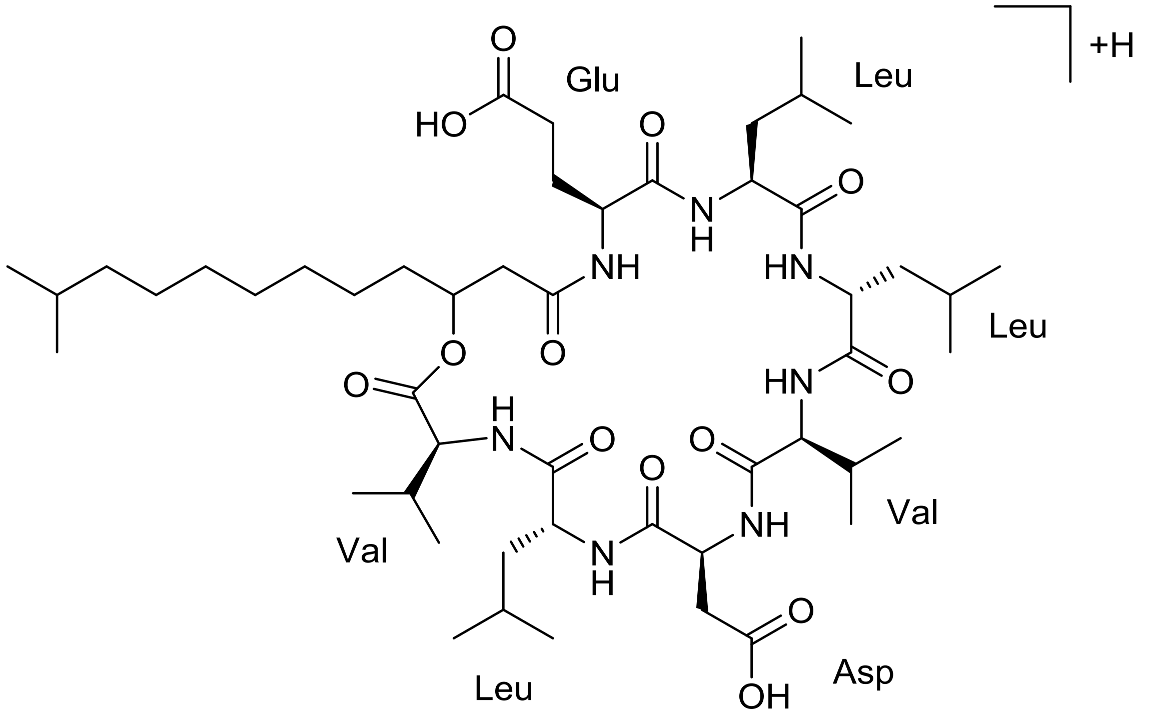

Surfactin B (C13)

Exact Mass: 994.6440

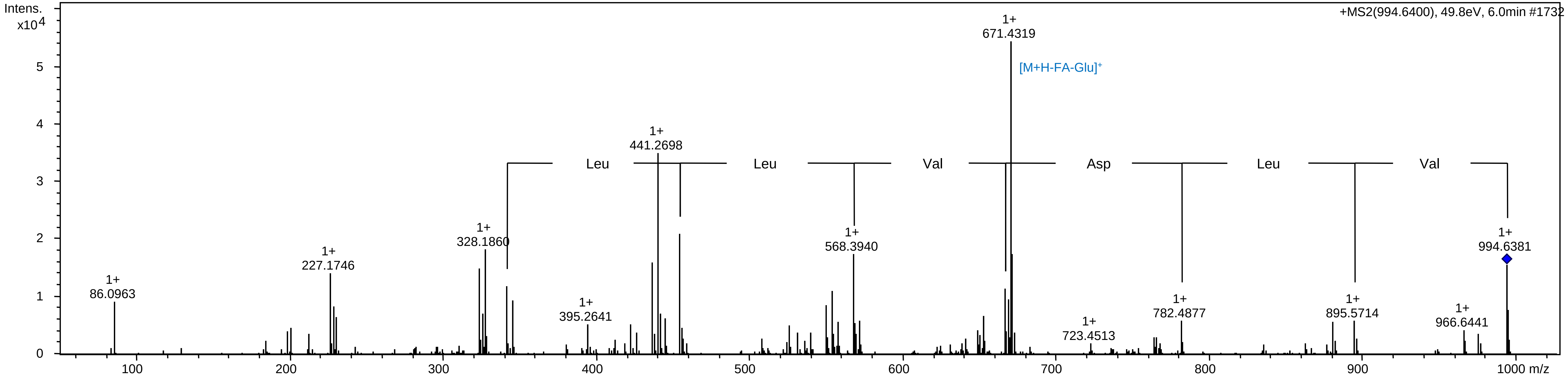

Figure S9. Surfactin B (C13). Tandem mass spectrometry (UHPLC-HRMS/MS) was used to identify the [M+H] ion at 5.94 ppm mass error. Identified fragments loss are depicted in the structure insert.

B. amyloliquefaciens MO4B

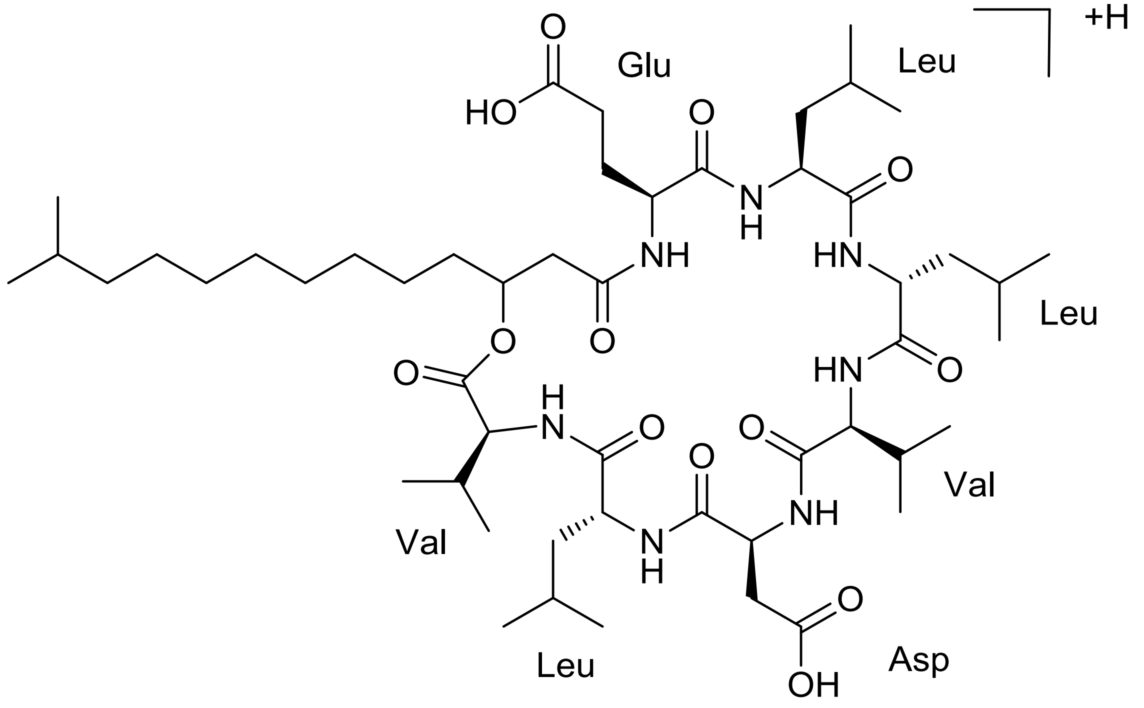

Surfactin B (C14)

Exact Mass: 1008.6597

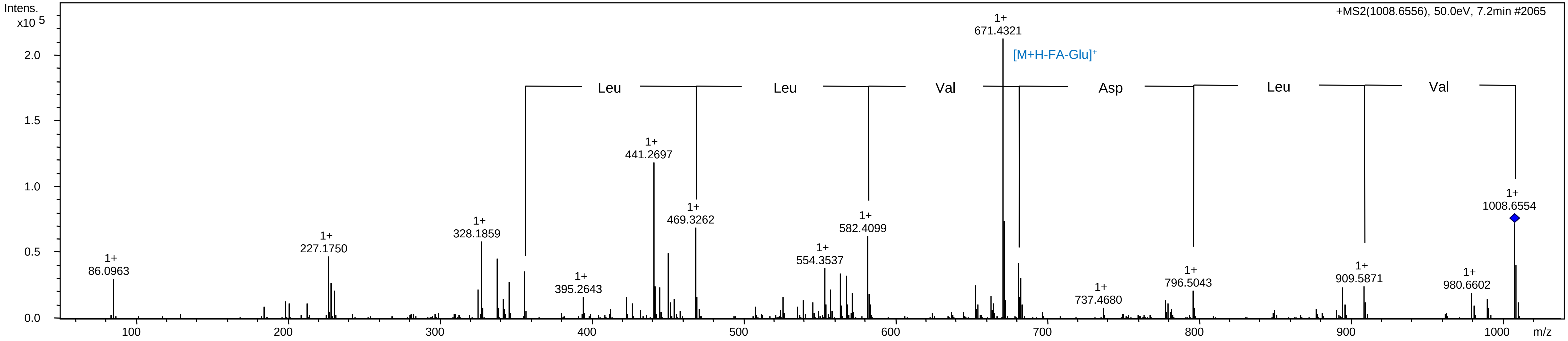

Figure S10. Surfactin B (C14). Tandem mass spectrometry (UHPLC-HRMS/MS) was used to identify the [M+H] ion at 4.22 ppm mass error. Identified fragments loss are depicted in the structure insert.

B. amyloliquefaciens MO4B

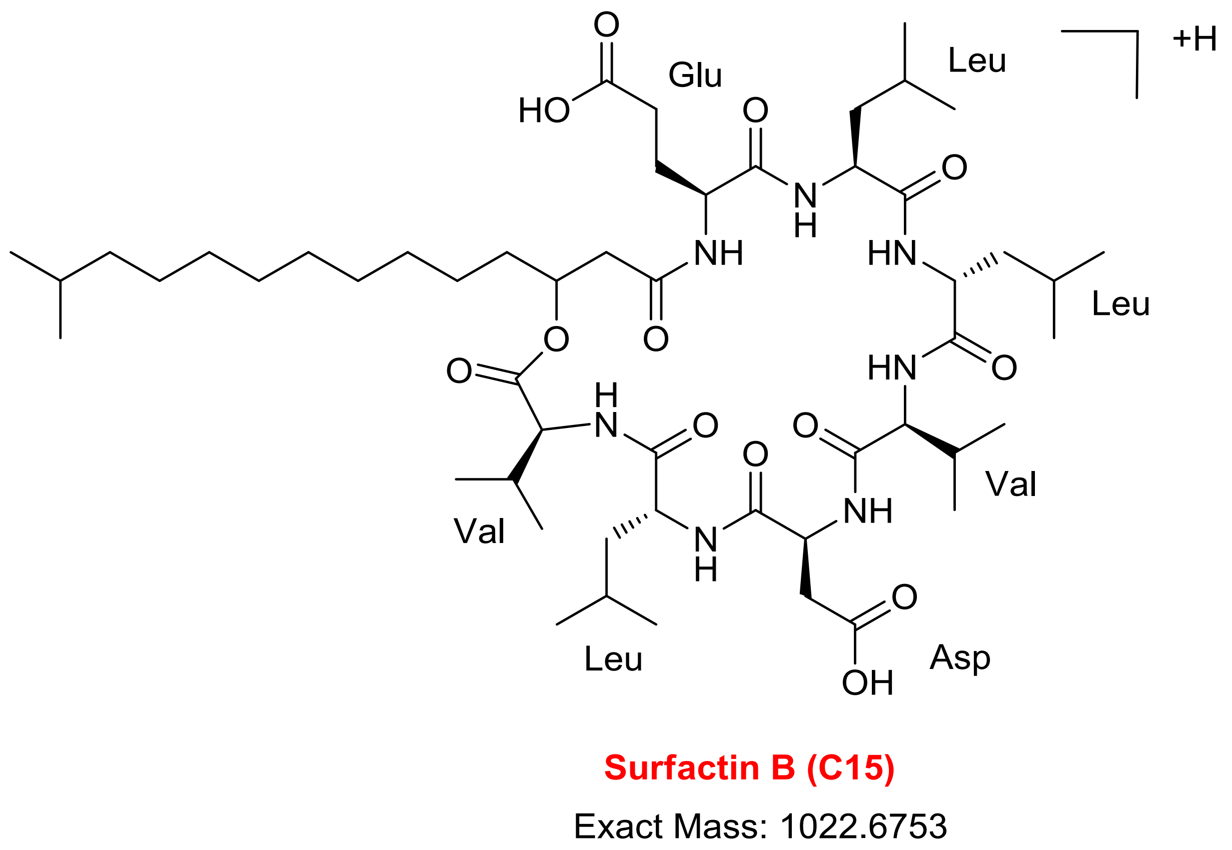

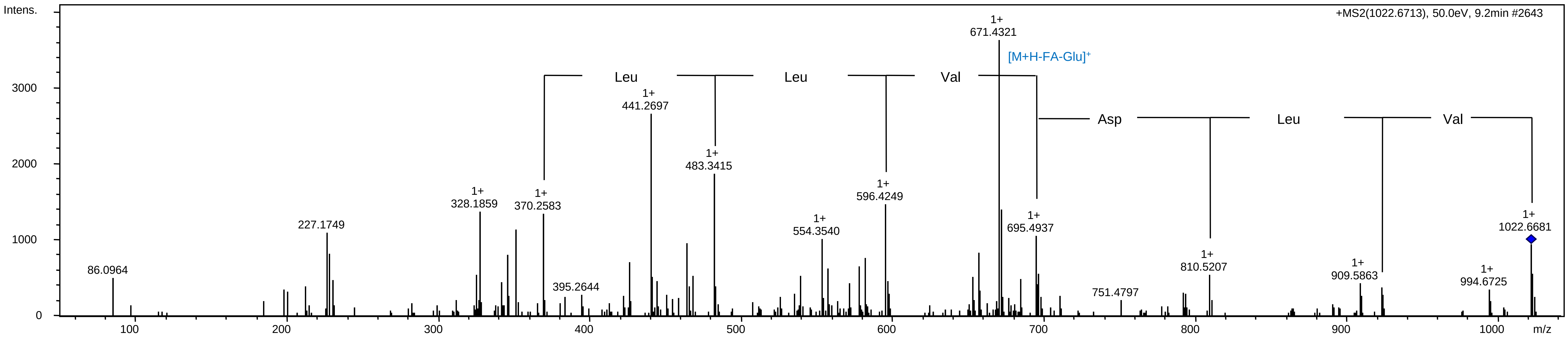

Figure S11. Surfactin B (C15). Tandem mass spectrometry (UHPLC-HRMS/MS) was used to identify the [M+H] ion at 3.92 ppm mass error. Identified fragments loss are depicted in the structure insert.

B. amyloliquefaciens MO4B

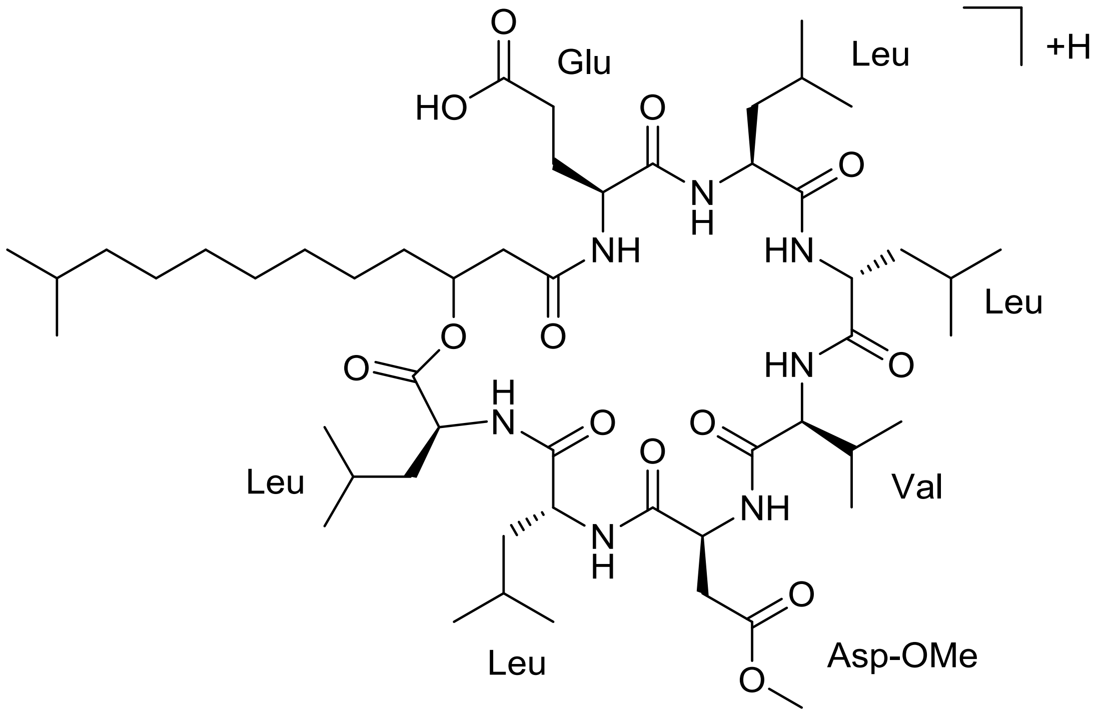

Surfactin monomethyl ester (C13)

Exact Mass: 1022.6753

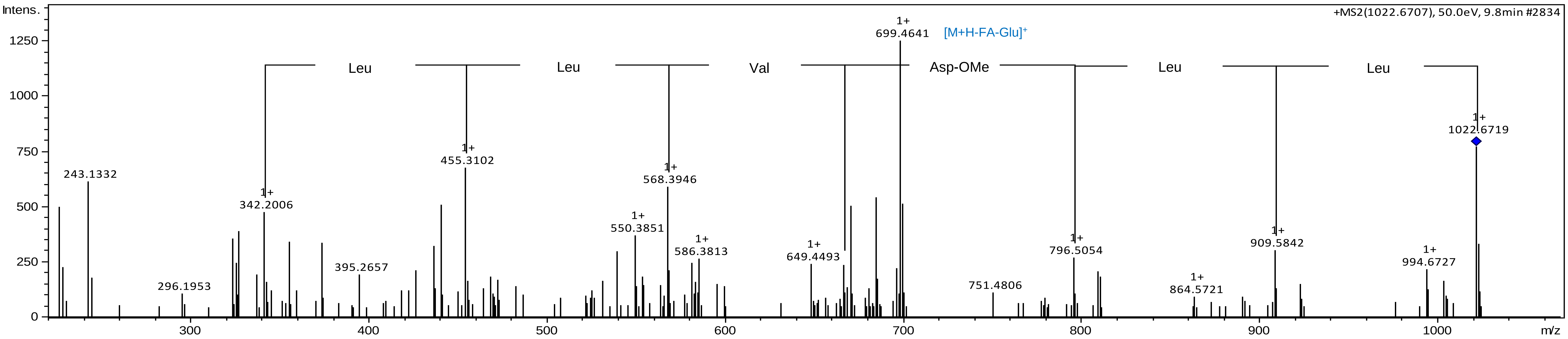

Figure S12. Surfactin monomethyl ester (C13). Tandem mass spectrometry (UHPLC-HRMS/MS) was used to identify the [M+H] ion at 3.34 ppm mass error. Identified fragments loss are depicted in the structure insert.

B. amyloliquefaciens MO4B

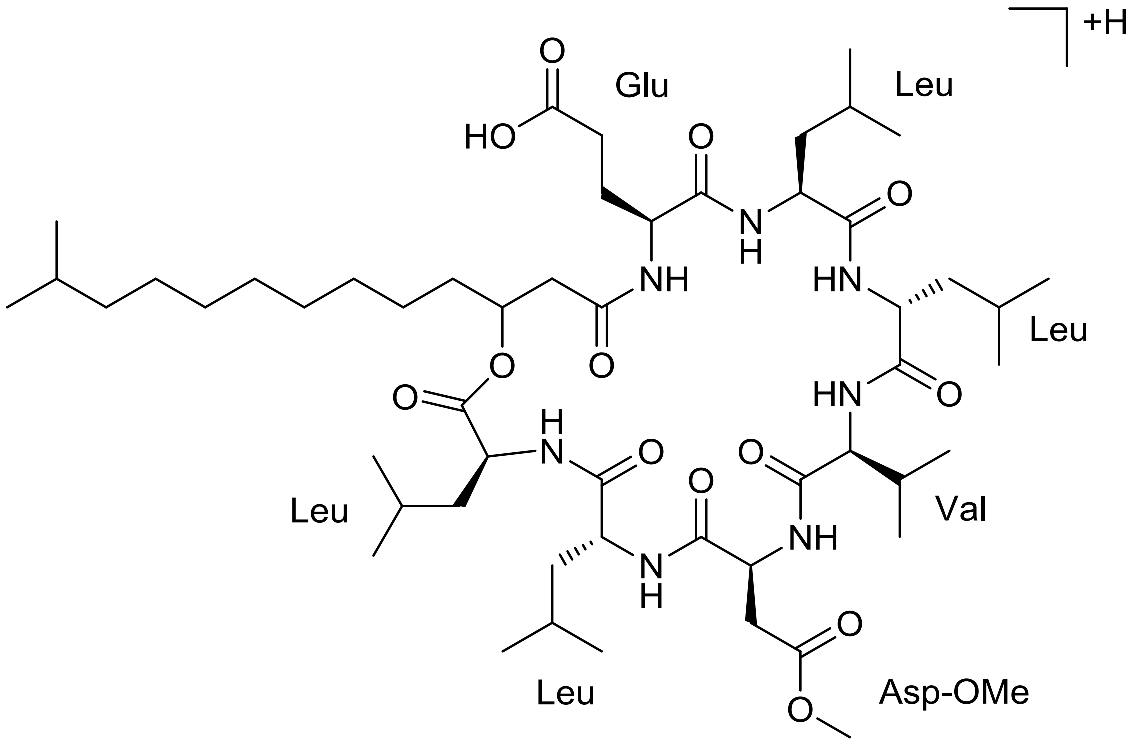

Surfactin monomethyl ester (C14)

Exact Mass: 1036.6910

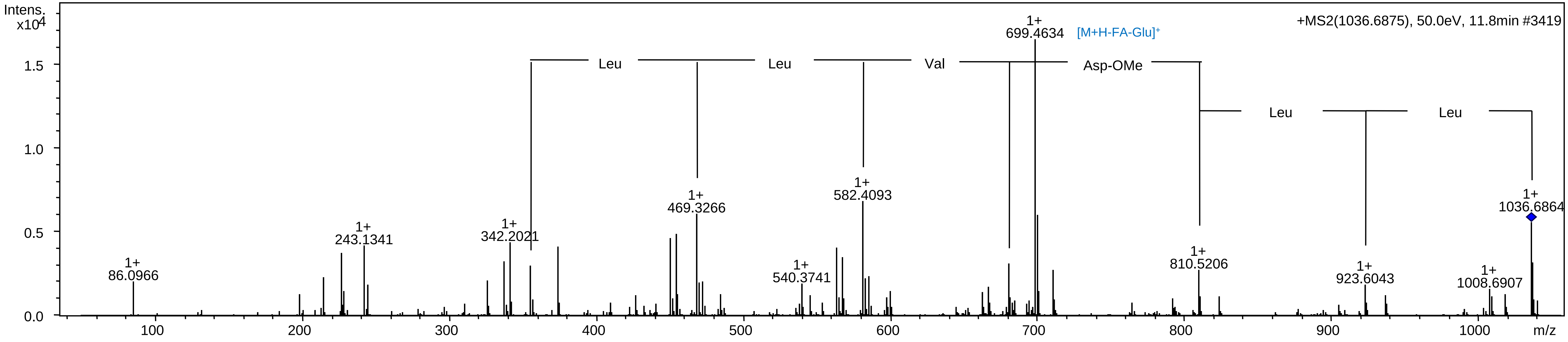

Figure S13. Surfactin monomethyl ester (C14). Tandem mass spectrometry (UHPLC-HRMS/MS) was used to identify the [M+H] ion at 4.40 ppm mass error. Identified fragments loss are depicted in the structure insert.

B. amyloliquefaciens MO4B

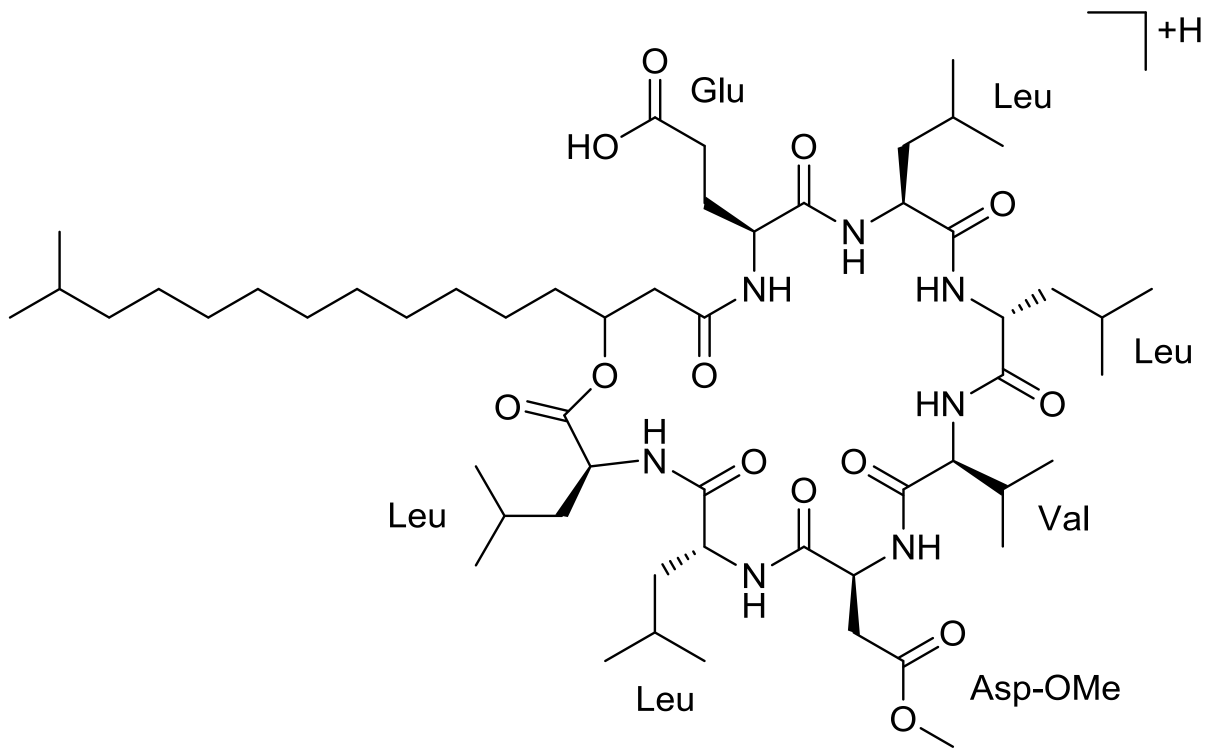

Surfactin monomethyl ester (C16)

Exact Mass: 1064.7223

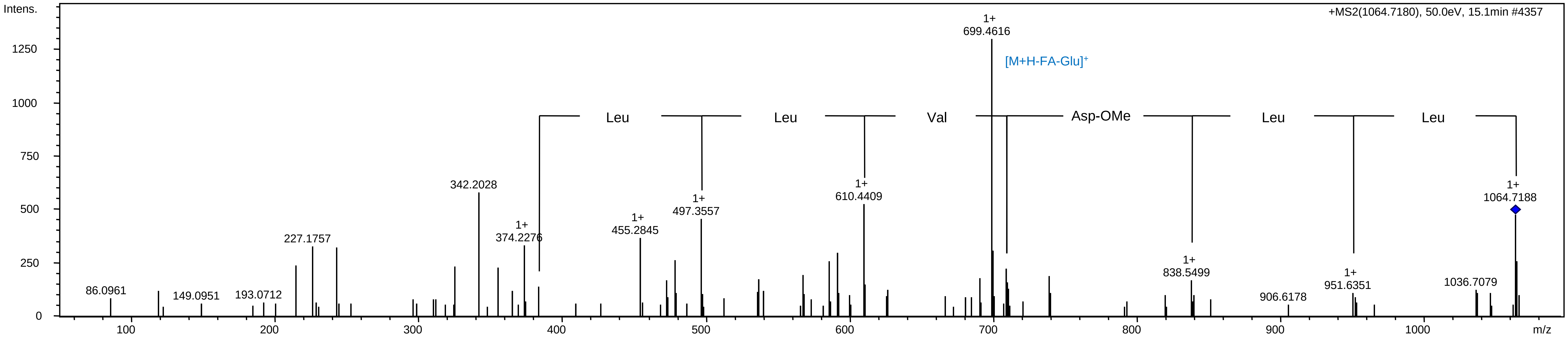

Figure S14. Surfactin monomethyl ester (C16). Tandem mass spectrometry (UHPLC-HRMS/MS) was used to identify the [M+H] ion at 3.25 ppm mass error. Identified fragments loss are depicted in the structure insert.

B. amyloliquefaciens MO4B

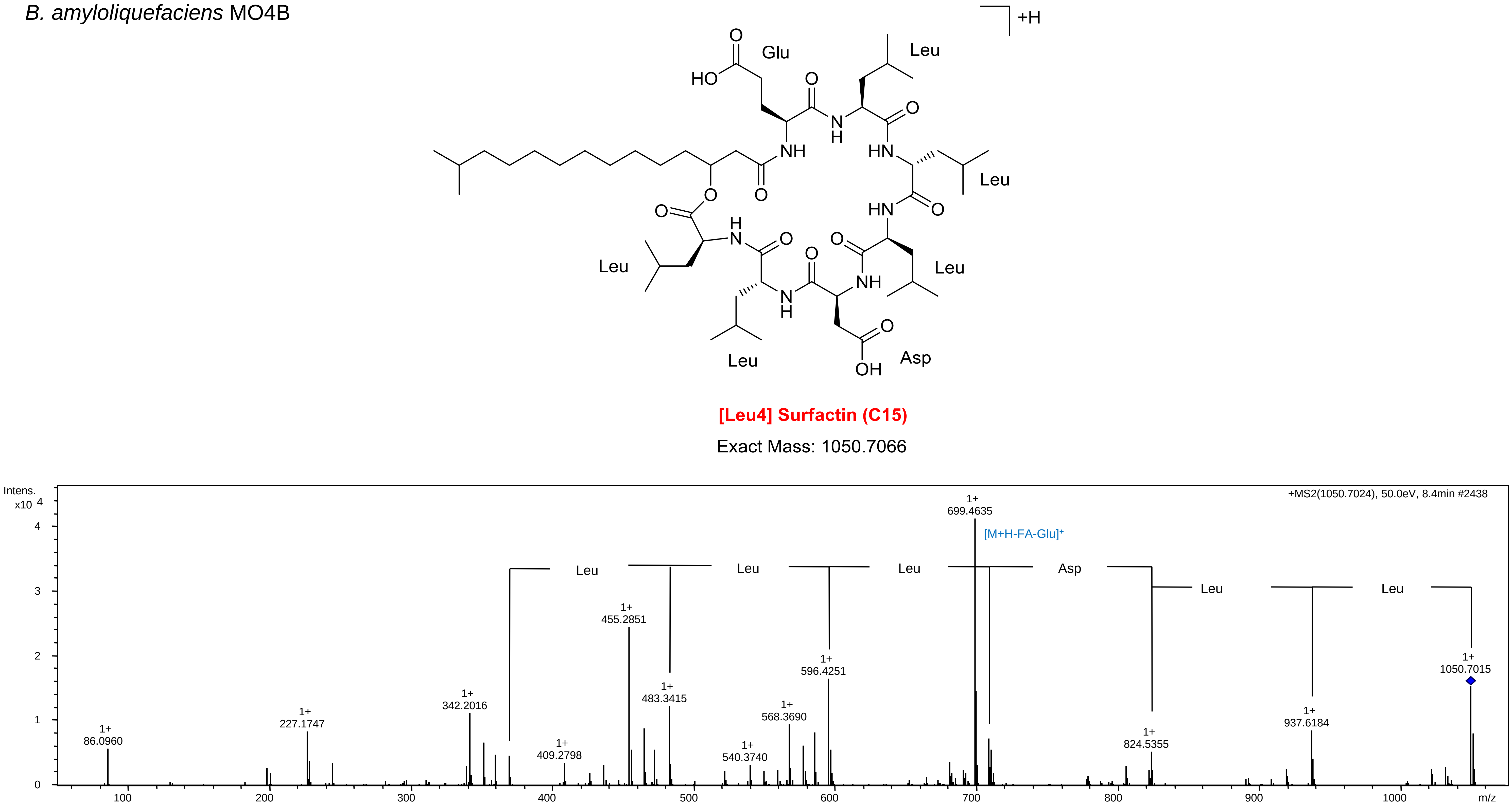

Figure S15. [Leu4] Surfactin (C15). Tandem mass spectrometry (UHPLC-HRMS/MS) was used to identify the [M+H] ion at 4.86 ppm mass error. Identified fragments loss are depicted in the structure insert.

B. amyloliquefaciens MO4B

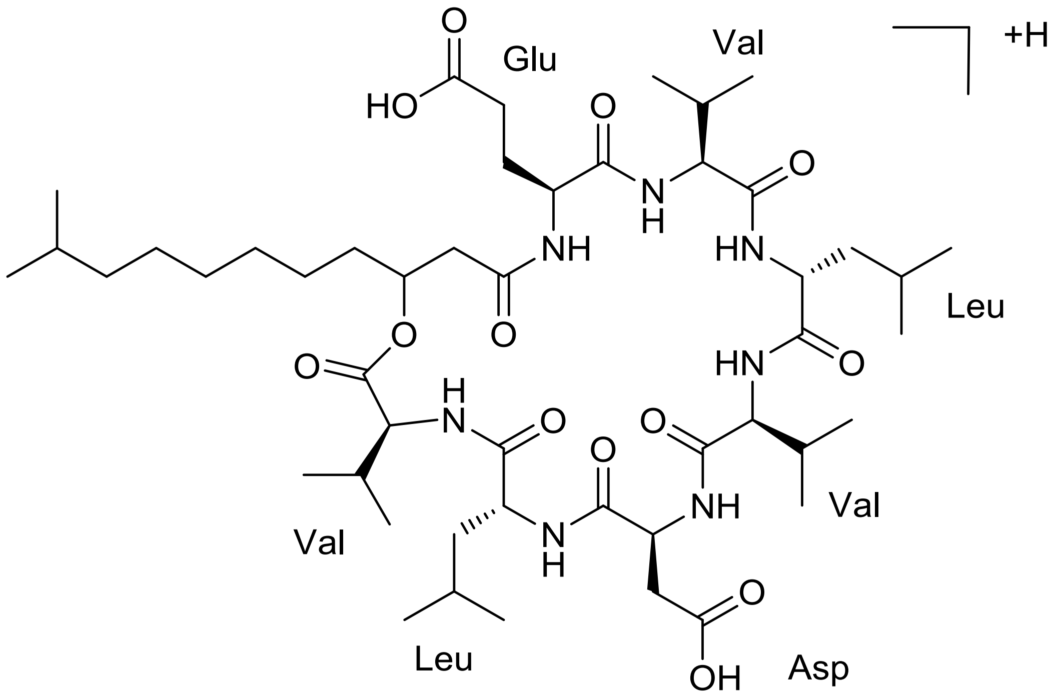

Surfactin isoform (C12)

Exact Mass: 966.6127

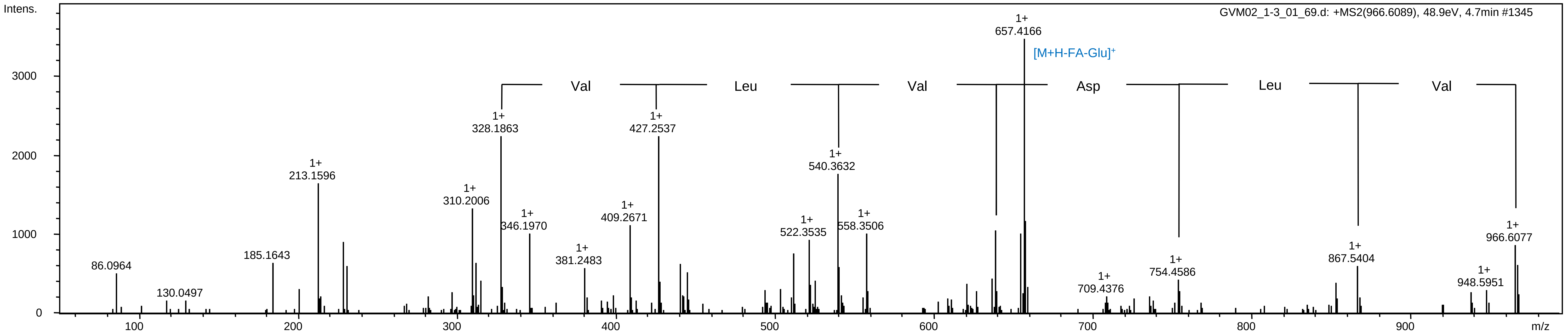

Figure S16. Surfactin isoform (C12). Tandem mass spectrometry (UHPLC-HRMS/MS) was used to identify the [M+H] ion at 5.18 ppm mass error. Identified fragments loss are depicted in the structure insert.

B. amyloliquefaciens MO4B

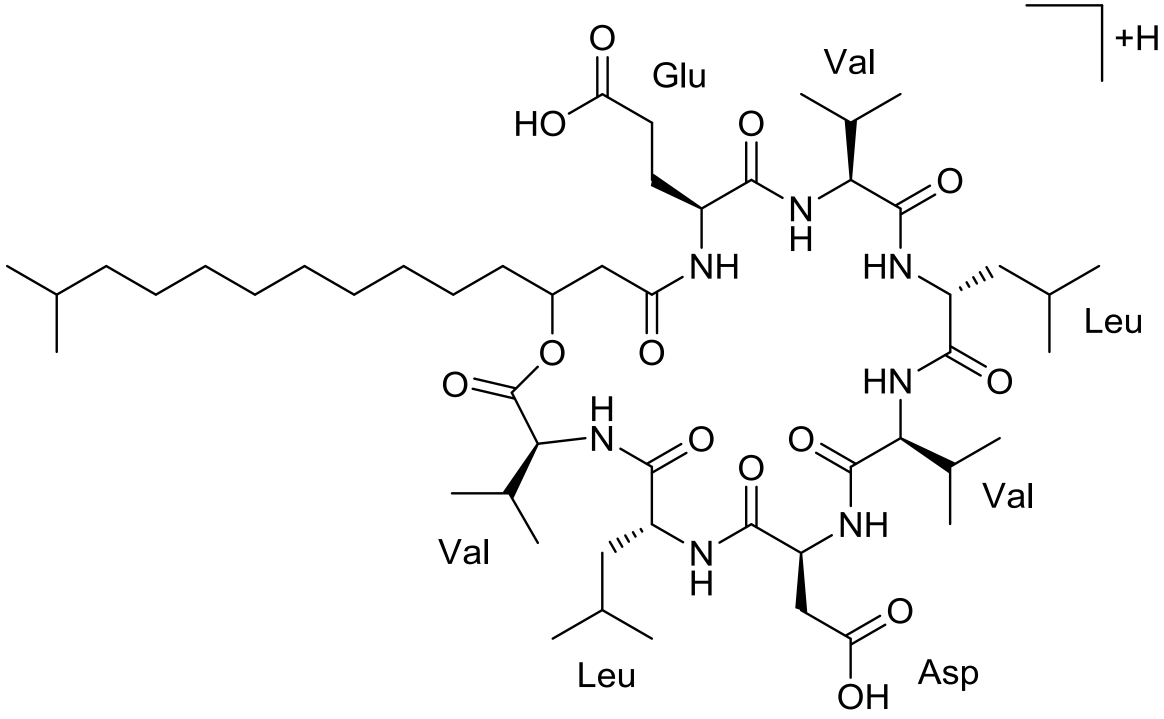

Surfactin Isoform (C15)

Exact Mass: 1008.6597

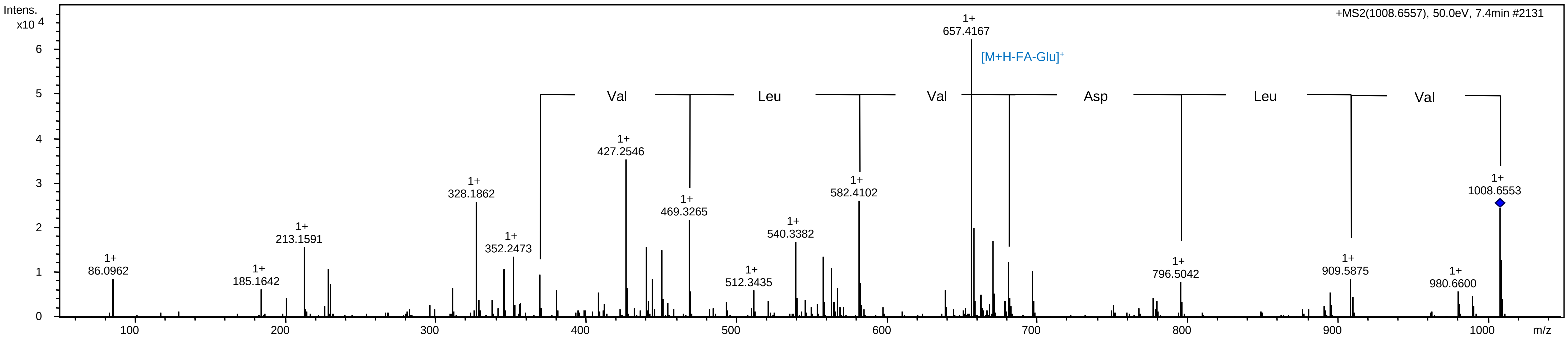

Figure S17. Surfactin isoform (C15). Tandem mass spectrometry (UHPLC-HRMS/MS) was used to identify the [M+H] ion at 4.32 ppm mass error. Identified fragments loss are depicted in the structure insert.

Table S1. Lipopeptide composition of *B. amyloliquefaciens* MO13 analyzed by UHPLC-ESI-MS/MS.

Structure	Molecular Formula	[M+H]+	Observed [M+H]+	Error (ppm)
**Surfactin A**				
C11	$C_{49}H_{85}N_7O_{13}$	980.628361	980.6245	3.94
C12	$C_{50}H_{87}N_7O_{13}$	994.644011	994.6394	4.64
C13	$C_{51}H_{89}N_7O_{13}$	1008.65966	1008.6555	4.13
C14	$C_{52}H_{91}N_7O_{13}$	1022.67531	1022.6719	3.34
C15	$C_{53}H_{93}N_7O_{13}$	1036.69096	1036.6874	3.44
C16	$C_{54}H_{95}N_7O_{13}$	1050.70661	1050.7019	4.48
C17	$C_{55}H_{97}N_7O_{13}$	1064.72226	1064.7178	4.19
**Surfactin B**				
C12	$C_{49}H_{85}N_7O_{13}$	980.628361	980.6237	4.75
C13	$C_{50}H_{87}N_7O_{13}$	994.644011	994.6399	4.13
C14	$C_{51}H_{89}N_7O_{13}$	1008.65966	1008.6557	3.93
C15	$C_{52}H_{91}N_7O_{13}$	1022.67531	1022.6717	3.53
**Surfactin monomethyl ester**				
C13	$C_{52}H_{91}N_7O_{13}$	1022.67531	1022.6724	2.85
C14	$C_{53}H_{93}N_7O_{13}$	1036.69096	1036.6869	3.92
C16	$C_{55}H_{97}N_7O_{13}$	1064.72226	1064.7175	4.47
**[Leu4] Surfactin**				
C15	$C_{54}H_{95}N_7O_{13}$	1050.70661	1050.7036	2.87
**Surfactin isoform**				
C15	$C_{51}H_{89}N_7O_{13}$	1008.65966	1008.6560	3.63

Table S2. Lipopeptide composition of *B. amyloliquefaciens* MO4B analyzed by UHPLC-ESI-MS/MS.

Structure	Molecular Formula	[M+H]+	Observed [M+H]+	Error (ppm)
**Surfactin A**				
C11	$C_{49}H_{85}N_7O_{13}$	980.628361	980.6229	5.57
C12	$C_{50}H_{87}N_7O_{13}$	994.644011	994.6401	3.93
C13	$C_{51}H_{89}N_7O_{13}$	1008.65966	1008.6560	3.63
C14	$C_{52}H_{91}N_7O_{13}$	1022.67531	1022.6703	4.90
C15	$C_{53}H_{93}N_7O_{13}$	1036.69096	1036.6869	3.92
C16	$C_{54}H_{95}N_7O_{13}$	1050.70661	1050.7020	4.39
C17	$C_{55}H_{97}N_7O_{13}$	1064.72226	1064.7187	3.34
**Surfactin B**				
C12	$C_{49}H_{85}N_7O_{13}$	980.628361	980.6245	3.94
C13	$C_{50}H_{87}N_7O_{13}$	994.644011	994.6381	5.94
C14	$C_{51}H_{89}N_7O_{13}$	1008.65966	1008.6554	4.22
C15	$C_{52}H_{91}N_7O_{13}$	1022.67531	1022.6681	7.05
**Surfactin monomethyl ester**				
C13	$C_{52}H_{91}N_7O_{13}$	1022.67531	1022.6719	3.34
C14	$C_{53}H_{93}N_7O_{13}$	1036.69096	1036.6864	4.40
C16	$C_{55}H_{97}N_7O_{13}$	1064.72226	1064.7188	3.25
**[Leu4] Surfactin**				
C15	$C_{54}H_{95}N_7O_{13}$	1050.70661	1050.7015	4.86
**Surfactin isoform**				
C12	$C_{48}H_{83}N_7O_{13}$	966.612711	966.6077	5.18
C15	$C_{51}H_{89}N_7O_{13}$	1008.65966	1008.6553	4.32

Table S3. Lipopeptide composition of *B. subtilis* ODW 02 analyzed by UHPLC-ESI-MS/MS.

Structure	Molecular Formula	[M+H]+	Observed [M+H]+	Error (ppm)
**Surfactin A**				
C12	$C_{50}H_{87}N_7O_{13}$	994.644011	994.6404	3.63
C13	$C_{51}H_{89}N_7O_{13}$	1008.65966	1008.6560	3.63
C14	$C_{52}H_{91}N_7O_{13}$	1022.67531	1022.6727	2.55
C15	$C_{53}H_{93}N_7O_{13}$	1036.69096	1036.6879	2.95
C16	$C_{54}H_{95}N_7O_{13}$	1050.70661	1050.7012	5.15
C17	$C_{55}H_{97}N_7O_{13}$	1064.72226	1064.7192	2.87
**Surfactin B**				
C13	$C_{50}H_{87}N_7O_{13}$	994.644011	994.6400	4.03
C14	$C_{51}H_{89}N_7O_{13}$	1008.65966	1008.6556	4.03
C15	$C_{52}H_{91}N_7O_{13}$	1022.67531	1022.6726	2.65
**Surfactin monomethyl ester**				
C14	$C_{53}H_{93}N_7O_{13}$	1036.69096	1036.6877	3.15
**[Leu4] Surfactin**				
C15	$C_{54}H_{95}N_7O_{13}$	1050.70661	1050.7028	3.63
**Surfactin isoform**				
C15	$C_{51}H_{89}N_7O_{13}$	1008.65966	1008.6569	2.74

Table S4. Lipopeptide composition *B. gibsonii* ODW12 analyzed by UHPLC-ESI-MS/MS.

Structure	Molecular Formula	[M+H]+	Observed [M+H]+	Error (ppm)
**Surfactin A**				
C12	$C_{50}H_{87}N_7O_{13}$	994.644011	994.6426	1.42
C13	$C_{51}H_{89}N_7O_{13}$	1008.65966	1008.6567	2.94
C14	$C_{52}H_{91}N_7O_{13}$	1022.67531	1022.6734	1.87
C15	$C_{53}H_{93}N_7O_{13}$	1036.69096	1036.6880	2.86
C16	$C_{54}H_{95}N_7O_{13}$	1050.70661	1050.7029	3.53
C17	$C_{55}H_{97}N_7O_{13}$	1064.72226	1064.7182	3.81
**Surfactin B**				
C13	$C_{50}H_{87}N_7O_{13}$	994.644011	994.6403	3.73
C14	$C_{51}H_{89}N_7O_{13}$	1008.65966	1008.6565	3.13
C15	$C_{52}H_{91}N_7O_{13}$	1022.67531	1022.6734	1.87
**Surfactin monomethyl ester**				
C14	$C_{53}H_{93}N_7O_{13}$	1036.69096	1036.6881	2.76
**[Leu4] Surfactin**				
C15	$C_{54}H_{95}N_7O_{13}$	1050.70661	1050.7034	3.06
**Surfactin isoform**				
C15	$C_{51}H_{89}N_7O_{13}$	1008.65966	1008.6566	3.03

Table S5. Lipopeptide composition *B. subtilis* ODW15 analyzed by UHPLC-ESI-MS/MS.

Structure	Molecular Formula	[M+H]+	Observed [M+H]+	Error (ppm)
**Surfactin A**				
C12	$C_{50}H_{87}N_7O_{13}$	994.644011	994.6414	2.63
C13	$C_{51}H_{89}N_7O_{13}$	1008.65966	1008.6555	4.13
C14	$C_{52}H_{91}N_7O_{13}$	1022.67531	1022.6731	2.16
C15	$C_{53}H_{93}N_7O_{13}$	1036.69096	1036.6885	2.37
C16	$C_{54}H_{95}N_7O_{13}$	1050.70661	1050.7036	2.87
C17	$C_{55}H_{97}N_7O_{13}$	1064.72226	1064.7196	2.50
**Surfactin B**				
C13	$C_{50}H_{87}N_7O_{13}$	994.644011	994.6410	3.03
C14	$C_{51}H_{89}N_7O_{13}$	1008.65966	1008.6580	1.65
C15	$C_{52}H_{91}N_7O_{13}$	1022.67531	1022.6719	3.34
**Surfactin monomethyl ester**				
C14	$C_{53}H_{93}N_7O_{13}$	1036.69096	1036.6878	3.05
**[Leu4] Surfactin**				
C15	$C_{54}H_{95}N_7O_{13}$	1050.70661	1050.7035	2.96
**Surfactin isoform**				
C15	$C_{51}H_{89}N_7O_{13}$	1008.65966	1008.6569	2.74
